# Intra-seasonal contrasting trends in clouds due to warming induced circulation changes

**DOI:** 10.1038/s41598-021-96246-2

**Published:** 2021-08-20

**Authors:** S. S. Prijith, C. B. Lima, M. V. Ramana, M. V. R. Sesha Sai

**Affiliations:** National Remote Sensing Centre, Indian Space Research Organisation, Hyderabad, 500037 India

**Keywords:** Climate sciences, Environmental sciences

## Abstract

Quantification of long term changes in cloud distribution and properties is critical for the proper assessment of future climate. We show contrasting trends in cloud properties and cloud radiative effects over Northwest Indian Ocean (NWIO) in south Asian summer monsoon. Cloud top height (CTH) decreases in June (− 69 ± 3 myr^−1^) and July (− 44 ± 3 myr^−1^), whereas it increases in August (106 ± 2 myr^−1^) and September (37 ± 1 myr^−1^). These contrasting trends are investigated to be due to the changes in upper tropospheric winds and atmospheric circulation pattern. Strengthening of upper tropospheric easterlies and changes in vertical wind dampen the vertical development of clouds in June and July. In contrast, weakening of upper tropospheric winds over NWIO and strengthening of updraft favour the vertical growth of clouds in August. Further, changes in horizontal winds at 450–350 hPa and strengthening of Indian Ocean Walker cell favour the westward spread of high level clouds, contributing to the increase in CTH over NWIO in August. Decrease of cloud cover and altitude in June and July and increase of the same in subsequent months would affect the monsoon rainfall over the Indian region. Proper representation of these intra-seasonal contrasting trends of clouds in climate models is important for the better prediction of regional weather.

## Introduction

Changes in amount and distribution of clouds, in a warming climate, assume importance as they modulate the shortwave and longwave cloud feedbacks^1,2^. By reflecting incoming shortwave radiation and trapping outgoing longwave radiation, clouds cause both cooling and warming in the Earth’s atmosphere^3–7^. However, net radiative effect of clouds is reported to be cooling, as the former one dominates^3,8–10^. Relative alterations in these shortwave and longwave radiative forcings, in response to climate change, determine the net cloud feedback^1,11,12^. While shortwave feedback is primarily due to the changes in cloud cover, longwave feedback mainly depends on the changes in cloud top height and temperature^13^. Net cloud radiative feedback is reported to be positive (0.6 Wm^−2^ ºC^−1^) by several models, though less certain, due to decrease in amount of low level clouds and increase in cloud altitudes^13,14^.

Changes in cloud distribution and associated alterations in cloud feedback form the largest source of uncertainty in future climate projections by the global climate models^13,15–19^. In order to minimise these uncertainties, it is essential to have better estimates of changes in cloud properties and distribution due to climate change^20^. Unfortunately, cloud variability and trends also remain substantially ambiguous on the global scale^21^. While the International Satellite Cloud Climatology Project (ISCCP) reports decreasing trends in total and high level clouds, there are other studies which show contrasting results^22,23^. Increase in high level clouds is reported over the tropics and northern hemisphere, but it is not seen to be so on the global scale^22^. Future climate projections by several model simulations show increase in cloud altitudes and resultant enhancement in cloud cover close to the tropopause^23,24^, due to deeper vertical motions in a warmer climate^25,26^. Increase in altitude of clouds leads to warming and hence to a positive feedback^27^. However, there are studies which suggest no change or slight increase in cloud top temperature (CTT) in a warmer climate^28,29^. Nevertheless, changes in horizontal spread and vertical development of clouds, due to warming induced modifications in atmospheric circulation patterns, can also lead to changes in regional and global mean cloud properties. Here, we show modifications in circulation patterns leading to contrasting trends in cloud properties, even within the same season, over the south Asian summer monsoon region.

## Results and discussion

### Trends in cloud properties over the south Asian summer monsoon region

**Figure 1:**
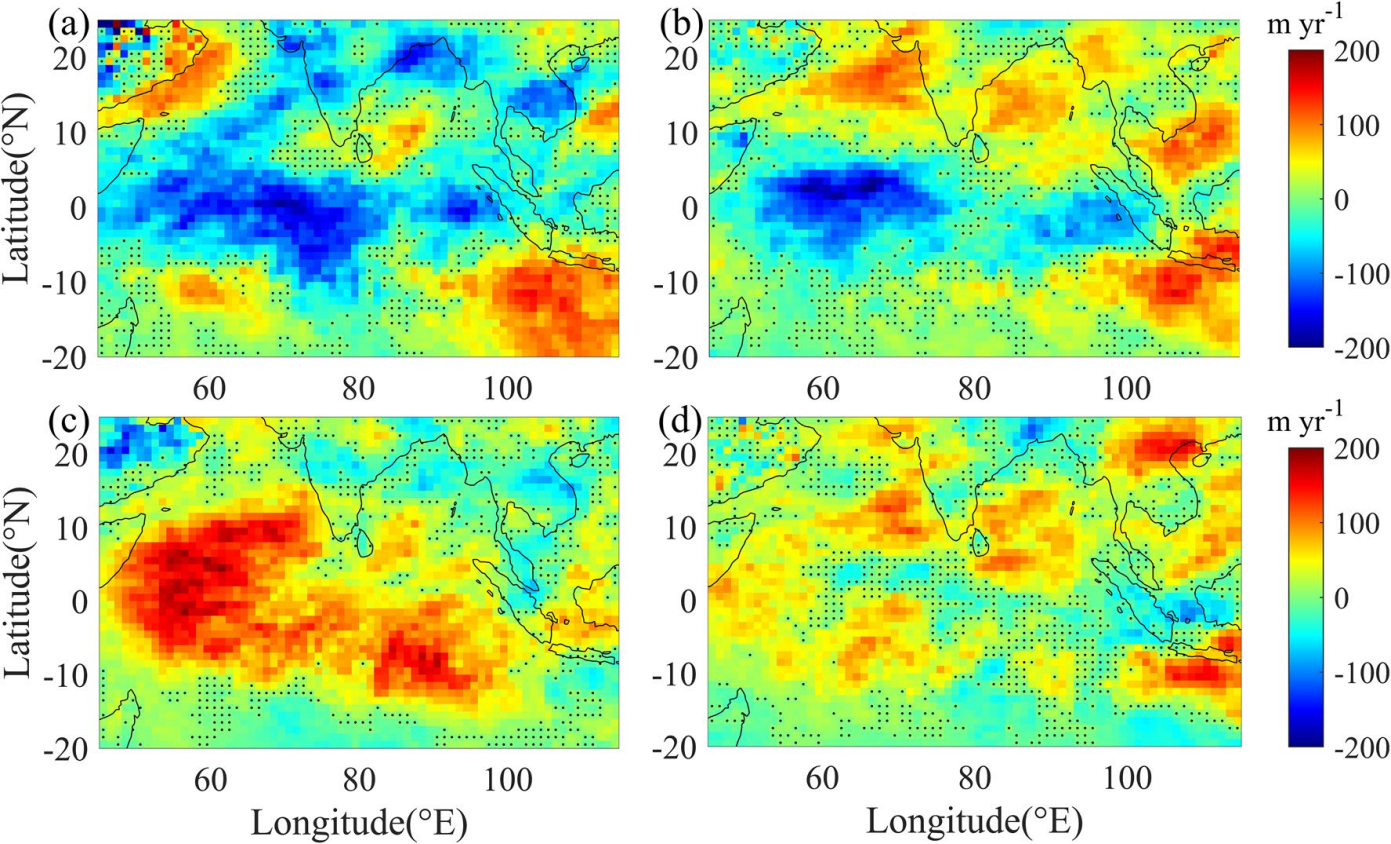
Spatial variations in trends of CTH in summer monsoon months: Spatial variations of trends in CTH from MODIS-Terra during 2000 to 2017, in the months of (**a**) June, (**b**) July, (**c**) August and (**d**) September. Positive values show the regions, where CTH is increasing and negative values indicate those, where CTH is decreasing. Linear trend of CTH in each pixel and its statistical significance are estimated using the trend analysis technique detailed in the method section. All the pixels, except those with black dots, show the regions where estimated trends are statistically significant at 95% confidence level. The map is generated using MATLAB 2020a, www.mathworks.com.

Tropical regions experience large amount of cloudiness, especially during the summer monsoon season (June–September). Hence, quantification of long term changes in cloud properties over the tropical regions is important to understand the climate change impacts on clouds and also to determine the cloud feedbacks. In summer monsoon period, cloud fraction (CF) over most of the regions of North Indian Ocean (NIO) is greater than 70% (see Supplementary Fig. S1a). However, cloudiness is more over the eastern parts of NIO, compared to that over the western parts. Cloud top height (CTH) is also observed to be higher over the former region, compared to that over the latter, indicating presence of clouds at higher altitudes over the eastern parts of NIO (see Supplementary Fig. S1b). Long term trends in CTH and CF over NIO in the summer monsoon months are estimated using the observations from MODIS-Terra during the period from 2000 to 2017 (see Fig. 1 and Supplementary Fig. S2). Our analysis shows contrasting trends in CTH and CF over the regions of northwest Indian Ocean (NWIO), even within the same season (summer monsoon). Decrease in CTH is observed in the first half of the season, along with a reduction in CF. In contrast, increase in CTH is seen together with an enhancement in CF in the second half of the season. Decreasing trend in CTH over NWIO (5° S to 15° N & 50° E to 75° E) is at the rate of − 69 ± 3 myr^−1^ and − 44 ± 3 myr^−1^ in June and July respectively (see Supplementary Table S1). Over the same region, CTH shows prominent positive trend in August, with rate of increase 106 ± 2 myr^−1^. However, rate of increase in September is observed to be 37 ± 1 myr^−1^. Trends in CF over NWIO are − 0.44 ± 0.02% yr^−1^, − 0.35 ± 0.01% yr^−1^, 0.3 ± 0.02% yr^−1^ and 0.03 ± 0.01% yr^−1^ in June, July, August and September respectively. Compared to NWIO, weaker trends in CTH are observed over the regions of northeast Indian Ocean (NEIO). Trend in CTH over NEIO (5° S to 15° N & 75° E to 100° E) is − 34 ± 2 myr^−1^ in June, 1 ± 2 myr^−1^ in July, 40 ± 2 myr^−1^ in August and 24 ± 2 myr^−1^ in September (see Supplementary Table S1). These trends show a total change in CTH of − 17%, − 11%, 30% and 12% over NWIO during the period from 2000 to 2017, in June, July, August and September respectively, with respect to the respective monthly mean CTH values during the period. However these changes over NEIO are only − 6.5% in June, 0.2% in July, 7.6% in August and 4.7% in September. Over the entire NIO region (5° S to 15° N & 50° E to 100° E), trends in CTH (CF) are observed to be − 51 ± 2 myr^−1^ (− 0.33 ± 0.01% yr^−1^), − 22 ± 2 myr^−1^ (− 0.19 ± 0.01% yr^−1^), 73 ± 2 myr^−1^ (0.24 ± 0.01% yr^−1^) and 30 ± 1 myr^−1^ (0.09 ± 0.01% yr^−1^) in June, July, August and September respectively. Trend in CTH over NWIO for the entire season is estimated to be 7 ± 1 myr^−1^, which is ~ 37% of the predicted rate of increase in upward shift of clouds (20 myr^−1^)^23^ over the tropics in a strong CO_2_ emission scenario^23^. Among all the four months of the season, strongest positive trend in CTH is observed in August, which is more than five times of the predicted rate of increase in CTH in the strong CO_2_ emission scenario. The contrasting trends in CTH over NWIO are observed to be consistent in both MODIS-Terra and MODIS-Aqua measurements. MODIS-Aqua measurements during 2002 to 2017 show trends in CTH of − 125 ± 3 myr^−1^, − 76 ± 3 myr^−1^, 84 ± 2 myr^−1^ and 53 ± 2 myr^−1^ over NWIO in June, July, August and September respectively.

**Figure 2:**
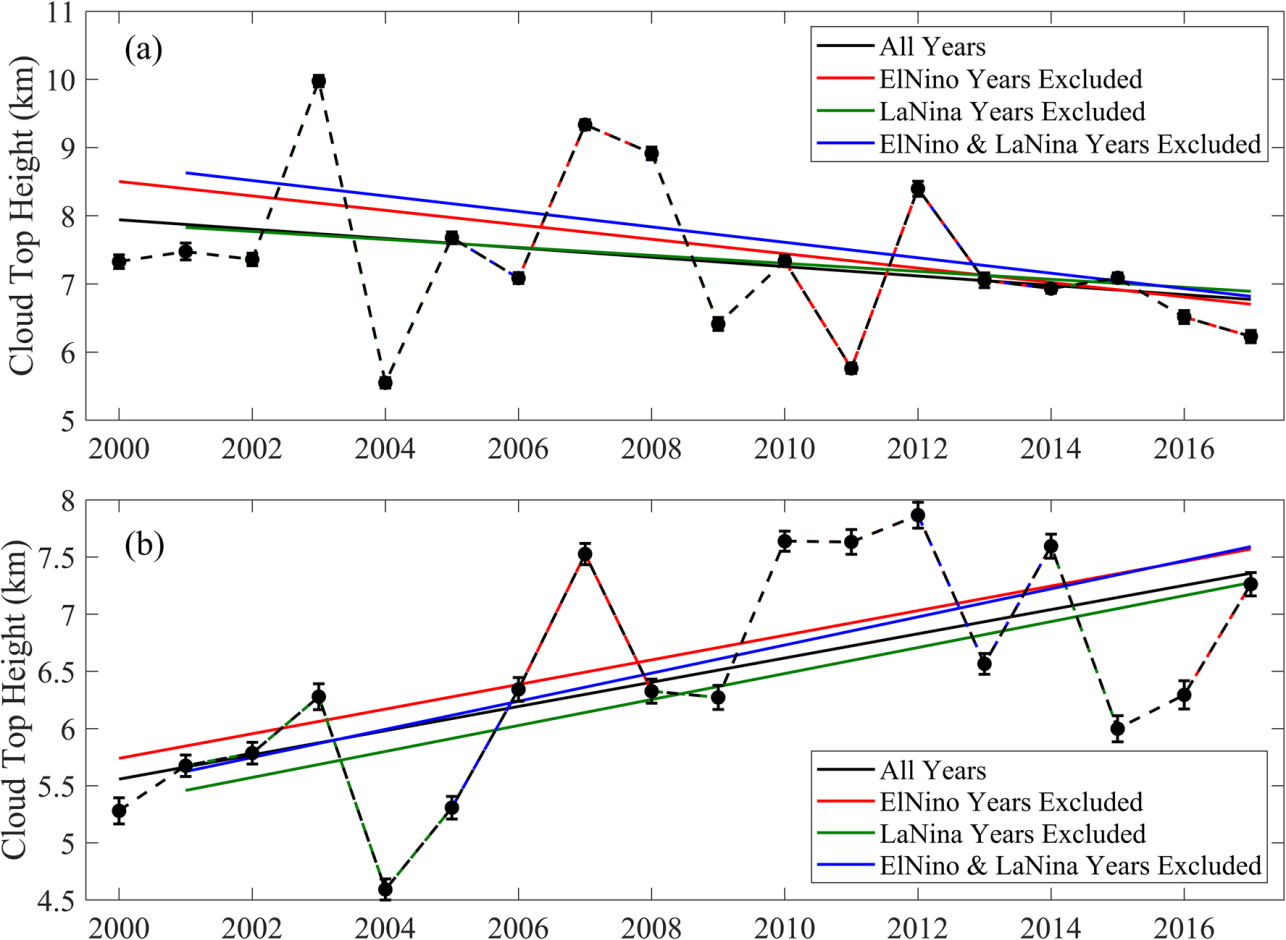
Effect of El Niño and La Niña in CTH trends: Mean CTH and its linear trend over the region, 5° S to 15° N & 50° E to 75° E, in the month of (**a**) June and (**b**) August, from MODIS-Terra measurements during 2000 to 2017. Black curves show the inter-annual variations and trends of CTH by considering all the years during the selected period, whereas red, green and blue curves show the same by excluding El Niño years, La Niña years and both El Niño and La Niña years. While filled circles show annual mean values of CTH, vertical bars represent corresponding standard errors. Solid lines in the figures show the estimated linear trends, for the four cases mentioned above.

In general, cloud distribution over the tropical regions is influenced by El Niño and La Niña events^30–32^. El Niño conditions decrease cloudiness over the regions of eastern Indian Ocean and western Pacific, whereas La Niña conditions enhance the convection activity and cloudiness over these regions^31,32^. In contrast, cloudiness over western regions of Indian Ocean enhances (diminishes) in El Niño (La Niña) conditions^31,32^. In order to examine the effects of these events, CTH trends in June and August are computed by including and excluding such events within the period of the study (see Fig. 2 and Supplementary Fig. S3). The months of June and August are considered as the representative months of first and second half of the season, as they experience the strongest negative and positive trends in CTH respectively. El Niño and La Niña years are identified using the Oceanic Niño Index (ONI)^33^ corresponding to the month of August (see “Methods” section). Contrasting trends in CTH over NWIO are more prominent, when the years of El Niño and La Niña events are excluded from the analysis (see Fig. 1 and Supplementary Fig. S3). In all the cases, decreasing trend of CTH in June and increasing trend in August are evident over the NWIO (see Fig. 2). When the years of El Niño and La Niña events are excluded, rate of decrease in CTH in June is − 113 ± 3 myr^−1^ and rate of increase in August is 123 ± 3 myr^−1^. The analysis confirms that the intra-seasonal contrasting trends in CTH over NWIO is factual and significant (see Supplementary Table S2), irrespective of the modifications due to the El Niño and La Niña events. Trends estimated by excluding El Niño and La Niña conditions show that the rate of decrease in CF is − 0.53 ± 0.02% yr^−1^ in June and − 0.64 ± 0.02% yr^−1^ in July, whereas the rate of increase is 0.51 ± 0.02% yr^−1^ and 0.07 ± 0.02% yr^−1^ in August and September respectively.

### Westward spread of high altitude clouds

**Figure 3:**
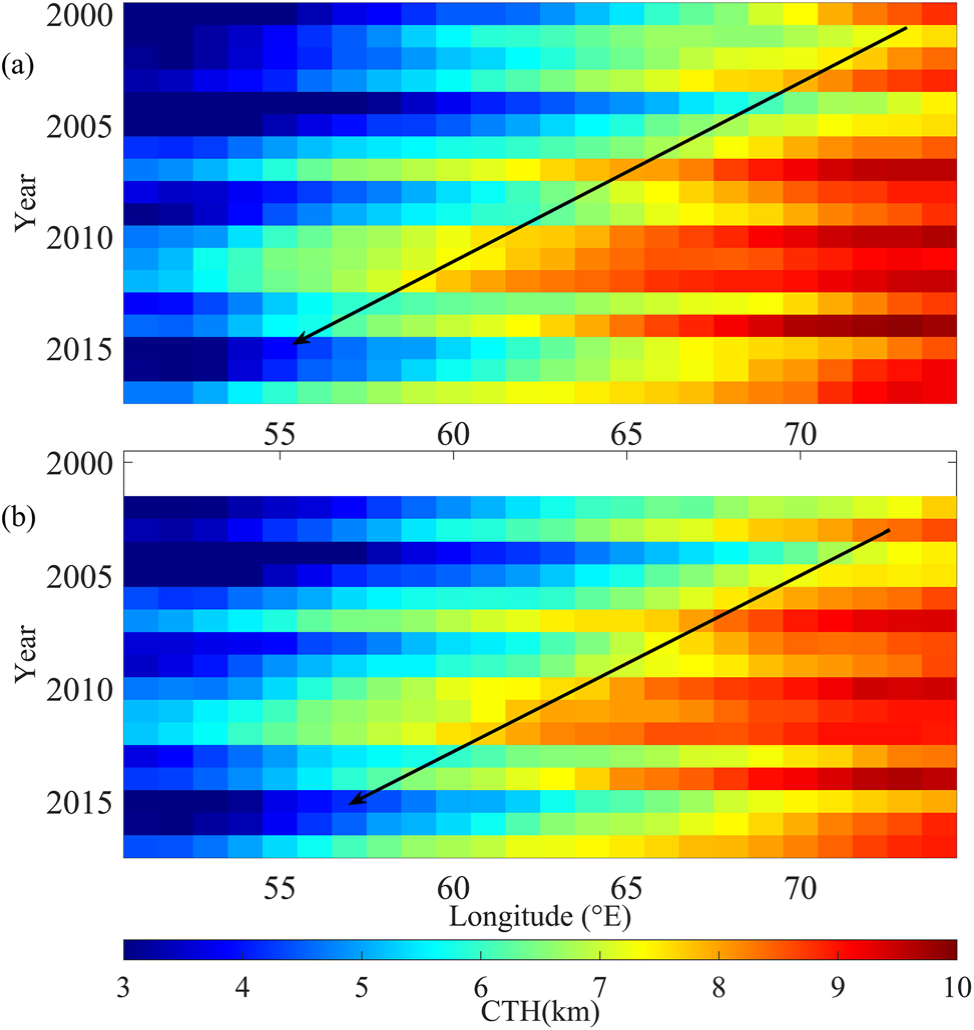
Westward spread of high level clouds: Cloud Top Height (CTH) in the month of August, averaged over the latitude band between 5° S and 15° N, from MODIS onboard (**a**) Terra and (**b**) Aqua, showing westward spread of high level clouds over north Indian Ocean. In general, CTH is higher over the eastern regions (as indicated by red color), compared to that over the western parts. Higher level clouds are observed to be spreading westward, over the years, as indicated by the black arrows.

Similar to the seasonal mean spatial distribution, clouds are seen at higher altitudes over the eastern regions of NIO, compared to those over the western regions, in the month of August. (see Supplementary Fig. S4). MODIS-Terra measurements during 2000 to 2017 show that the mean CTH in August over NWIO is 6.46 ± 0.03 km and that over NEIO is 9.48 ± 0.01 km. This difference in cloud altitudes between the western and eastern regions of NIO is corroborated by the results from MODIS-Aqua measurements during 2002 to 2017, which show mean CTH of 6.42 ± 0.03 km and 9.29 ± 0.01 km in August over the NWIO and NEIO respectively. Mean CTH over NWIO in August is observed to be increasing, from ~ 5.5 km to more than 7 km over the years (see Fig. 2b). MODIS-Terra measurements show that the increase in CTH over NWIO during 2000 to 2017 is 34%, with respect to the mean value during the period, whereas that over NEIO is merely 9%. MODIS-Aqua measurements also show consistent results of 29% increase in CTH over NWIO and 7% increase over NEIO during 2002 to 2017. Further, mean CTH in August, averaged over the latitude band between 5° S and 15° N, shows westward spread of high level clouds over NIO during 2000 to 2017 (see Fig. 3). Mean CF averaged over the same latitude band also shows the westward spread of clouds over this region (see Supplementary Fig. S5). High level clouds are found mainly over the regions at east of 65°E in the initial years, whereas they spread westwards and seen up to the regions of 60°E in the final years of the study period. Further, we identified the mean longitude, within the latitude band between 5° S and 15° N, with clouds at east of it at higher altitudes (CTH > 7 km, corresponding to ~ 440 hPa) and those at west of it at lower altitudes. This mean longitude is represented as LON_7km_, hereafter in this manuscript. LON_7 km_ is observed to be drifting westward over the years, from ~ 66° E in 2000 to ~ 60° E in 2017 (see Supplementary Fig. S6). Shift of LON_7km_ shows that clouds with CTH above 7 km are mainly seen over the regions at east of 66° E in 2000 and their westward spread leads to the presence of high level clouds even over the western regions, up to ~ 60° E, in 2017. This westward spread of high level clouds, over the years, leads to the increasing trends in CTH and CF over the NWIO. In general, northward migration of monsoon cloud-bands from the equatorial Indian Ocean trough region to inland plays a major role in the monsoon rainfall over India. Hence the decrease of CF and CTH in June and July over NWIO and increase of the same in subsequent months would affect the precipitation over the Indian region.

### Physical mechanisms

#### Influence of upper tropospheric winds on cloud distribution

**Figure 4:**
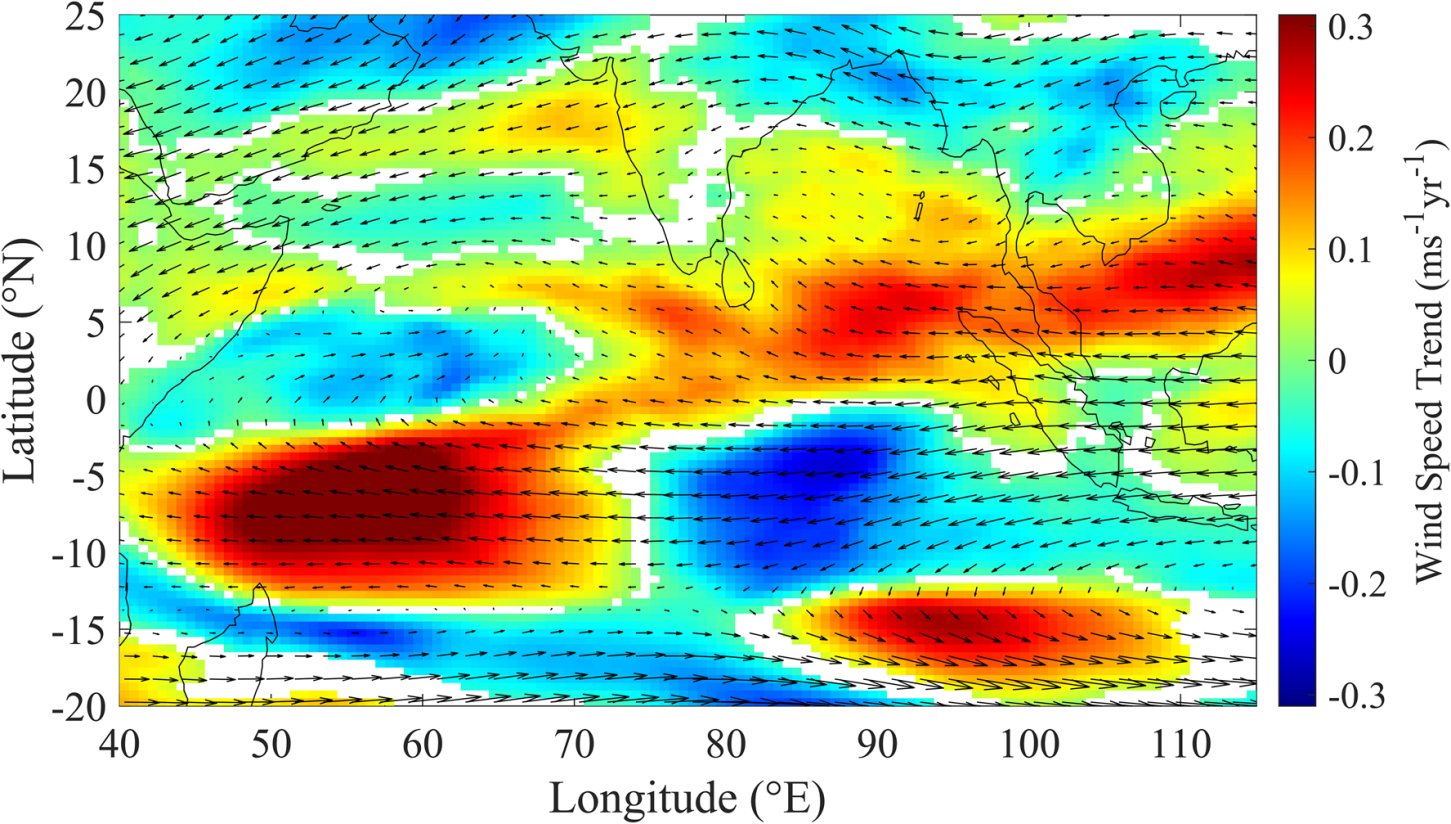
Trends of horizontal winds at 450–350 hPa: Trends of horizontal wind speed (represented by color) at altitudes of 450 to 350 hPa, in the month of August during 2000–2017, excluding the years of El Niño and La Niña conditions. Black arrows embedded in the figure indicate the mean wind pattern during the same period. Positive (negative) values indicated by colors show the regions where winds are strengthening (weakening), whereas white patches show the regions where trends are not statistically significant at 95% confidence level. The map is generated using MATLAB 2020a, www.mathworks.com.

Role of higher altitude winds in the westward spread of high level clouds is examined by estimating trends of horizontal wind speed at the altitude levels corresponding to 450 to 350 hPa (see Fig. 4). Strengthening of winds is observed in August over the regions at west of 75° E, extending westward up to 40° E, within the latitude band between 13° S and 3° S. Within the latitude band between 3° S and 5° N, increase in wind speed is seen over the regions at east of 75° E. It is interesting to notice the weakening of winds over the regions of NWIO, where increase in CTH is observed. Over the southern hemispheric part, easterlies are observed to be turning over the region between 40° E and 60° E and becoming southwesterlies towards the regions of increase in CTH. This leads to sweeping of more clouds from the regions of increase in wind speed, over the southern hemispheric part, to the regions where CTH is found to be increasing. Easterlies over the northern hemispheric part (between equator and 5° N) also push more clouds to the west, from the regions where they are strengthening.

Cloud distribution over the south Asian summer monsoon region is highly influenced by the Tropical Easterly Jet (TEJ) stream^34,35^. These strong easterly winds sweep clouds at higher altitudes and hence inhibit vertical growth of clouds over this region beyond an altitude limit (~ 300 hPa)^34^. In order to examine the changes in upper tropospheric winds, trends of wind speed at 200 hPa in the summer monsoon months are examined (see Supplementary Fig. S7). Estimated trends show strengthening of upper tropospheric winds over the NIO at south of the core regions of TEJ, in June and July. This indicates increase in hindrance to vertical growth of clouds over this region in these months, which leads to the negative trends in CTH. However, decreasing trends in wind speed are seen over the NWIO in August, which favour further vertical development of clouds over this region.

#### Changes in convection and Indian Ocean Walker Cell

**Figure 5:**
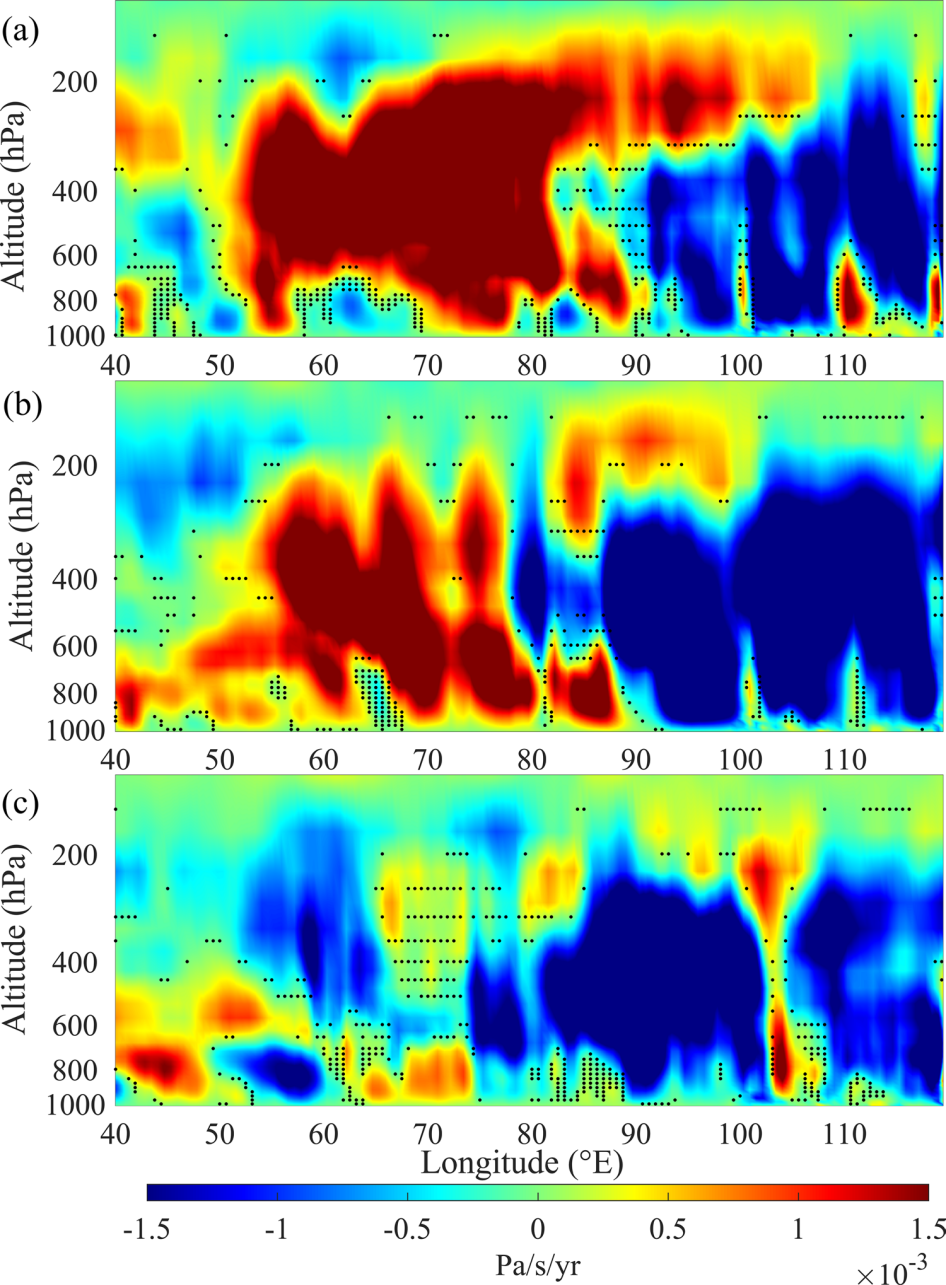
Trends of vertical winds in summer monsoon months: Trends of vertical wind averaged over the latitude band between 10° S and equator, in (**a**) June, (**b**) July and (**c**) August during 2000–2017, by excluding El Niño and La Niña years. In order to maintain uniformity, El Niño and La Niña years are considered based on the ONI values corresponding to August. Black dots show the regions where trends are not significant at 95% confidence level. Positive values indicate strengthening (weakening) of downward (upward) wind and negative values indicate vice versa.

In general, NIO experiences strong updraft over the eastern parts (east of 80° E) and downdrafts over the western regions (west of 60° E), as indicated by the mean vertical winds over this region in the summer monsoon months (see Supplementary Fig. S8). Long term changes in convection over NIO are examined by estimating trends of vertical winds, averaged over the latitude band between 10° S and equator, in June, July and August during 2000 to 2017, by excluding El Niño and La Niña events (see Fig. 5). Exclusion of El Niño and La Niña years is done based on the ONI values corresponding to the month of August, only to maintain uniformity among all the three months. Positive values show the regions where downdraft is strengthening or updraft is weakening, whereas negative values indicate the regions where updraft is strengthening or downdraft is weakening. Intensification of upward winds is observed over the eastern regions of NIO in all the three months. However, weakening of updrafts (or strengthening of downdrafts) is observed over the regions between 55° E and 80° E in June and July. In contrast, trends of vertical winds show strengthening of updraft (or weakening of down draft) over the regions between 55° E and 65° E, at the altitude levels extending from 600 hPa to above 200 hPa, in August. These contrasting trends in vertical winds favour further vertical development of clouds over this region in August and prevent the same in the previous two months, leading to increase in CTH in August and decrease in CTH in June and July. The trends in vertical winds are in agreement with the changes in upper tropospheric horizontal winds over this region (see Supplementary Fig. S7). Upper tropospheric horizontal winds show decreasing trend in August, which favours increase in updraft and vertical developments of clouds. In contrast, upper tropospheric winds strengthen and hence inhibit the vertical updraft in June and July.

Indian Ocean Walker circulation cell is strengthening in summer monsoon months, as indicated by the enhancement in updraft over the regions at east of 80° E and strengthening of downdraft over the regions at west (see Fig. 5). These changes in vertical winds favour intensification of upper level easterlies over this region, as the Indian Ocean Walker cell is characterised by upper level easterlies and lower level westerlies along with the updraft and downdraft over the eastern and western regions respectively. However, the weakening of updrafts (or the strengthening of downdrafts) over the regions between 55° E and 80° E in June and July prevents further westward spread of clouds over to the regions at west of 55° E. Compared to these months, strengthening of downdraft in August is observed further west, over the region between 40° E and 50° E. This favours further westward spread of high level clouds in August, leading to the increase in CTH over NWIO. Strengthening of the Indian Ocean Walker circulation cell in the month of August is further examined by considering long term changes of difference in mean vertical wind over the ascending and descending limbs of Indian Ocean Walker cell. Ascending (Descending) limb of the Indian Ocean Walker cell in August can be seen over the regions between 80° E and 100° E (40° E and 60° E) (see Supplementary Fig. S8c). Changes in the strength of the Indian Ocean Walker cell are estimated by considering the difference in mean vertical wind, over the regions 45° E–55° E (descending limb) and 85° E–95° E (ascending limb), at the altitude levels from 600 to 400 hPa for the latitude band between 10° S and equator. Increasing trend of difference in vertical wind between west and east indicates strengthening of the Walker circulation cell over this region (see Supplementary Fig. S9). This strengthening of the Walker circulation cell over the Indian Ocean is reported to be in response to the warming climate^36,37^. This leads to strengthening of surface level westerlies over the equatorial Indian Ocean and enhancement in upward air motion over its eastern parts^37^. This enhancement in convection over the eastern parts of the equatorial Indian Ocean (see Fig. 5) and associated strengthening of easterly winds at higher altitudes lead to westward spread of high level clouds. Strengthening of convection over the eastern regions causes increase in CTH over NEIO also. However, intensification of upper tropospheric easterlies (Supplementary Fig. S7) inhibits further vertical development of clouds over this region. Warming of the tropical Indian Ocean is reported to be primarily due to the increase in concentrations of greenhouse gases and associated enhancement in downward longwave radiation^38^. This, along with the results presented here, indicates the effect of anthropogenic climate warming on atmospheric circulation and hence on cloud distribution over the south Asian summer monsoon region. Convection activity over the eastern tropical Indian Ocean weakens during El Niño events, whereas it strengthens during La Niña. These variations in strength of convection and associated changes in upper level easterlies lead to modifications in the magnitude of observed trends in CTH over the NWIO (see Fig. 2).

Westward spread of high level clouds in August reduces the difference in CTH, existed among the summer monsoon months, over NWIO. Mean CTH values over NWIO from MODIS-Terra during 2000 to 2009 and MODIS-Aqua during 2002 to 2009, are compared with those during the latter period from 2010 to 2017. Mean CTH in August during the former period is considerably lower than that in the other two months. Mean CTH in August during 2000 to 2009 (2002 to 2009) is 5.94 ± 0.03 km (5.99 ± 0.04 km) from Terra (Aqua) measurements, whereas it is 7.71 ± 0.03 km (7.74 ± 0.04 km) and 7.39 ± 0.03 km (7.43 ± 0.04 km) in June and July respectively. However, mean CTH is almost same (~ 7 km) in all the three months during the latter period. Compared to the former period, latter period shows decrease in CTH in June and July.

Climate conditions over the equatorial Indian Ocean are affected by the Pacific Decadal Oscillation (PDO), which is a dominant pattern of natural climate variability in the Pacific Ocean at decadal to multi-decadal time scales^39,40^. PDO is reported to affect the circulation pattern, including the Indian Ocean Walker cell and upper tropospheric winds, over this region^41^. While cold phase of the PDO supports the normal pattern of Indian Ocean Walker cell with convection over the eastern regions of equatorial Indian Ocean, warm phase leads to a contrasting pattern with subsidence over the same region^41^. During the current study period, PDO shifted between its warm and cold phases (see Supplementary Fig. S10a). In order to examine the effect of PDO, the trends of CTH in August are examined separately for the warm and cold phases of PDO. Though the rate of increase in CTH is less in the warm phase, compared to that in the cold phase, positive trend in CTH is observed irrespective of the phase changes of PDO (see Supplementary Fig. S10b). Thus, the increasing trend in CTH over the NWIO is persistent, with only changes in the magnitude of rate of increase, in spite of the alterations in climate conditions due to PDO.

## Impact on cloud top temperature

The intra-seasonal contrasting changes in clouds lead to increase in CTT over NWIO in the first half of the season and decrease of CTT in the second half (see Supplementary Fig. S11). MODIS-Terra (MODIS-Aqua) observations show increase in mean CTT over NWIO at a rate of 0.48 ± 0.02 Kyr^−1^ (0.87 ± 0.02 Kyr^−1^) in June. However, westward spread of high level clouds leads to a significant decrease in CTT over the NWIO in August. Decrease in CTT is observed to be at a rate of − 0.7 ± 0.01 Kyr^−1^ (− 0.54 ± 0.01 Kyr^−1^) in August, from MODIS-Terra (MODIS-Aqua) observations. The rate of increase of CTT in June is 0.78 ± 0.02 Kyr^−1^ (1.24 ± 0.02 Kyr^−1^) and the rate of decrease in August is − 0.81 ± 0.02 Kyr^−1^ (− 0.67 ± 0.02 Kyr^−1^), from MODIS-Terra (MODIS-Aqua) measurements, when the years of El Niño and La Niña events are excluded from the analysis. The increasing trend of CTT indicate increase in outgoing longwave radiation in the first half of the season, whereas the decreasing trend in CTT indicate decrease in outgoing longwave radiation over this region in the second half of the season.

## Changes in top of the atmosphere (TOA) fluxes and cloud radiative effects (CRE)

The observed changes in cloud properties over NWIO lead to alterations in TOA fluxes and CRE (see Supplementary Fig. S12 and Supplementary Tables S3 and S4). During the study period from 2000 to 2017, mean shortwave cloud radiative effect (CRE_SW_) and longwave cloud radiative effect (CRE_LW_) over NWIO in the first half of the season are − 45.78 Wm^−2^ and 46.96 Wm^−2^ respectively, which make the net cloud radiative effect (CRE_NET_) over this region 1.19Wm^−2^. However, in the second half of the season, mean CRE_SW_, CRE_LW_ and CRE_NET_ are − 41.73 Wm^−2^, 37.57 Wm^−2^ and − 4.15 Wm^−2^ respectively. CRE_LW_ dominates CRE_SW_ in June and July and hence results into a net warming (positive CRE_NET_) in the first half of the season, whereas CRE_SW_ dominates CRE_LW_ in August and September and hence leads to a net cooling (negative CRE_NET_) in the second half. However, seasonal mean CRE_SW_ (− 43.75 Wm^−2^) dominates CRE_LW_ (42.27 Wm^−2^) in summer monsoon period and hence results into a net cooling (CRE_NET_ of − 1.48 Wm^−2^) over NWIO, which is in agreement with the global net cloud radiative effect^8–10^. Decrease in cloud cover leads to a change of 15.33Wm^−2^ in CRE_SW_ and decrease in CTH causes a change of − 17.98 Wm^−2^ in CRE_LW_ the first half of the season. In contrast, increase in cloud cover changes CRE_SW_ by − 9.89 Wm^−2^ and increase in CTH changes CRE_LW_ by 9.57Wm^−2^ in the second half. Thus, the changes in cloud properties diminish CRE_SW_ and CRE_LW_ (positive ΔCRE_SW_ and negative ΔCRE_LW_) in the first half of the season, whereas they enhance both CRE_SW_ and CRE_LW_ (negative ΔCRE_SW_ and positive ΔCRE_LW_) in the second half. While ΔCRE_SW_ is higher (15.33 Wm^−2^) in the first half of the season, opposite ΔCRE_SW_ (− 9.89 Wm^−2^) in the second half reduces the seasonal mean CRE_SW_ to 2.72 Wm^−2^. Similarly, while first half of the season experiences a larger CRE_LW_ of − 17.98 Wm^−2^, contrasting CRE_LW_ of 9.57Wm^−2^ in the second half makes the seasonal mean CRE_LW_ − 4.21 Wm^−2^. Thus, the larger changes in both shortwave and longwave radiative effects observed in the first half of the season are opposed by the contrasting changes in the second half of the season. Hence, the change in seasonal mean is CRE_NET_ is − 1.49 Wm^−2^ over NWIO, whereas it is − 2.65 Wm^−2^ in the first half of the season. Though changes in clouds lead to a larger cooling trend in the first half, opposite changes in the second half reduces the magnitude of the seasonal mean cooling trend over NWIO. These alterations in cloud properties lead to contrasting changes in TOA shortwave and longwave fluxes (F_SW_ and F_LW_) in the former and latter half of the season. While decrease in TOA F_SW_ and increase in TOA F_LW_ are observed under all sky conditions in June and July, opposite changes are seen in the fluxes in August and September (see Supplementary Table S3). Changes in TOA F_SW_ are − 14.89 Wm^−2^ and 9.76 Wm^−2^ in the first and second half of the season respectively under all sky conditions, whereas the changes are merely 0.44 Wm^−2^ and − 0.13 Wm^−2^ under clear sky conditions. Changes in TOA outgoing F_LW_ under all sky conditions are observed to be 19.31 Wm^−2^ and − 12.57 Wm^−2^ in the first and second half of the season respectively, whereas those under clear sky conditions are 1.33 Wm^−2^ and − 3.00 Wm^−2^ respectively. Larger changes in TOA fluxes in the first half of the season is opposed by the contrasting changes in the second half and hence results into a decrease in seasonal mean TOA F_SW_ of − 2.56 Wm^−2^ and increase in seasonal mean TOA F_LW_ of 3.37 Wm^−2^ under all sky conditions.

## Discussion

Increase in global temperature has significant effect on the hydrological cycle and cloud distribution, especially over the tropical regions. Cloud feedback in response to the global temperature increase is determined by the net effect of its shortwave and longwave feedbacks. Alterations in cloud cover and altitude affect both of these feedbacks. Our study shows changes in atmospheric circulation pattern and their effects on cloud distribution over the south Asian summer monsoon region. Alterations in atmospheric circulation pattern leading to contrasting trends in cloud properties, even within the same season, show the importance of proper incorporation of these changes in climate models. Intensification of upper tropospheric easterlies and weakening of updraft (strengthening of downdraft) increase hindrance to the vertical development of clouds and hence decrease CTH over NWIO in the first half of the south Asian summer monsoon period. In contrast, weakening of the upper tropospheric easterlies over NWIO and strengthening of updraft provide favourable condition for the vertical developments of clouds and hence increase CTH in the second half of the season. Along with this, changes in horizontal winds at 450 to 350 hPa (~ 7 km to 9 km) altitude levels lead to spread of clouds at these levels to the regions of NWIO. Warming induced intensification of the Indian Ocean Walker cell and associated strengthening of upper level easterlies favour the westward spread of high level clouds, leading to the increase in CTH and CF over the NWIO in August. However, weakening of updraft (strengthening of downdraft) over the regions between 55° E and 80° E prevents further westward spread of high level clouds in the first half of the season. Strengthening of downdraft in August occurs further west, over the regions between 40° E and 50° E, which causes further westward spread of high level clouds compared to that in the previous months. This enhancement in westward movement of clouds leads to increase in amount of high level clouds and hence increase in mean CTH and CF over NWIO in the second half of the season, whereas the hindrance to this westward movement of clouds causes decrease in amount of high level clouds and hence decrease in mean CTH and CF over NWIO in the first half. Thus the changes in atmospheric circulation lead to contrasting trends in cloud height and cloud cover over NWIO in south Asian summer monsoon, with decrease in CTH (− 69 ± 3 myr^−1^ in June and − 44 ± 3 myr^−1^ in July) and CF (− 0.44 ± 0.02% yr^−1^ in June and − 0.35 ± 0.01% yr^−1^ in July) in the first half of the season and increase in CTH (106 ± 2 myr^−1^ in August and 37 ± 1 myr^−1^ in September) and CF (0.3 ± 0.02% yr^−1^ in August and 0.03 ± 0.01% yr^−1^ in September) in the second half. Impact of these intra-seasonal contrasting changes of clouds on the south Asian summer monsoon rainfall needs to be examined. NWIO experiences a net warming in the first half of the season as CRE_LW_ dominates CRE_SW_, whereas the region experiences a net cooling in the second half as CRE_SW_ dominates CRE_LW_. However, the seasonal mean CRE_NET_ is observed to be negative, indicating a net cooling over the NWIO. The alterations in cloud properties lead to contrasting changes in both CRE_SW_ and CRE_LW_ in the first and second half of the season. While decrease in cloud cover changes CRE_SW_ by 15.33Wm^−2^ and decrease in CTH changes CRE_LW_ by − 17.98 Wm^−2^ in the first half, increase in CF and CTH lead to a change of − 9.89 Wm^−2^ and 9.57 Wm^−2^ in CRE_SW_ and CRE_LW_ respectively in the second half. While changes in clouds lead to a stronger cooling trend in the first half of the season, contrasting changes in the second half weaken the seasonal mean cooling trend over NWIO. Alterations in atmospheric circulation pattern and associated modifications in cloud distribution are more likely to occur over several other regions in a warming climate scenario. Global scale analysis needs to be carried out to estimate the net effect of these changes on the cloud feedback. As cloud cover is a crucial factor for the sensitivity of general circulation models^24,42^, capability of proper representations of these intra-seasonal contrasting trends in clouds is decisive for the realistic predictions of regional weather phenomena such as south Asian summer monsoon and associated rainfall.

## Methods

### MODIS cloud parameters

Trends of cloud properties are estimated using collection 6.1(C6.1), L3 data of monthly mean CTH, CF and CTT at a spatial resolution of 1° × 1°, from the Moderate Resolution Imaging Spectroradiometer (MODIS) onboard both Terra and Aqua satellites. MODIS retrieves cloud top pressure (CTP), using CO_2_ slicing technique and an alternate method with brightness temperature at 11μm^43,44^. CO_2_ slicing method makes use of the variation in radiation absorption within the broad 15 μm CO_2_ absorption band^44^. CO_2_ slicing technique is mostly suitable for mid level to high level clouds, whereas the latter one is more effective in the presence of low level clouds with CTP greater than 700hPa^43^. Retrieved CTP is converted to CTH and CTT, using gridded temperature profiles from National Centers for Environmental Prediction Global Data Assimilation System^45,46^. Thus, CTH from MODIS is the geopotential height at the retrieved cloud top pressure level. Collection 6 (C6) MODIS cloud top products have improved, compared to its previous versions, in several aspects due to the refinements in algorithm and calibration^47,48^. The calibration approaches employed in the generation of C6 products remove the calibration trends in the data^49^. In comparison with Cloud-Aerosol Lidar with Orthogonal Polarization (CALIOP) measurements, C6 MODIS CTH shows a bias of 197 m, for low level clouds^47^. However, there are issues associated with C6 products, due to the electronic crosstalk contamination, which are corrected in C6.1 data products^50^. MODIS C6.1 data sets used in the present study are available through https://ladsweb.modaps.eosdis.nasa.gov.

### Trend analysis

Trend analysis is carried out by estimating linear trends of the parameters and corresponding uncertainty in each pixel^51,52^. Significance of the estimated trends is assessed using the values of absolute trend relative to corresponding uncertainty. In order to estimate the trends, a linear model is considered as Y_t_ = μ + ωX_t_ + N_t_, t = 1, 2, 3, …., n, where Y_t_, ω, X_t_, n and N_t_, are the parameter, trend, time in year, number of years and noise respectively. Further, statistical significance of the estimated trends is assessed using absolute values of the ratio of trend to corresponding uncertainty $\left( {\left| {\frac{\omega }{{\sigma_{\omega } }}} \right|} \right)$. σ_ω_ is calculated as

1 $$\sigma_{\omega } = \frac{{\sigma_{N} }}{{n^{{\left( {3/2} \right)}} }}\sqrt {\frac{1 + \phi }{{1 - \phi }}} ,$$

where σ_N_ is standard deviation and ϕ is autocorrelation coefficient of noise. If the value of $\left| {\frac{\omega }{{\sigma_{\omega } }}} \right|$ is greater than 2, corresponding trend (ω) is considered to be statistically significant at 95% confidence level^53^.

### MERRA2 reanalysis

In order to investigate the role of atmospheric dynamics, horizontal and vertical components of winds are used from Modern-Era Retrospective analysis for Research and Applications Version 2 (MERRA2) reanalysis, by NASA’s Global Modeling and Assimilation Office (GMAO). MERRA2 is advanced, compared to MERRA, in terms of assimilation of more satellite observations in the reanalysis^54^. In order to assimilate newer satellite observations, MERRA2 uses an upgraded version of Goddard Earth Observing System Model (GEOS-5), compared to that used in MERRA reanalysis^55^. MERRA2 provides data of atmospheric parameters at a spatial resolution of 0.5° × 0.625° at 72 pressure levels from surface to an altitude corresponding to 0.01hPa^54^. MERRA2 data set used in the present study is obtained through https://disc.gsfc.nasa.gov.

### Oceanic Niño Index

Oceanic Niño Index (ONI) is one of the parameters used to identify El Niño and La Niña conditions and quantify the strength of ENSO. ONI is estimated as the three month running average of sea surface temperature anomaly over the Niño-3.4 region (5° S–5° N, 120° W–170° W)^33^. Thus, ONI value corresponding to the month of August is the running mean of the anomaly for the three months from July to September. Conditions with ONI ≥ 0.5 °C is considered as El Niño event and that with ONI ≤  − 0.5 °C is considered as La Niña^56^. Thus 2002, 2004, 2009 and 2015 are considered as El Niño years, whereas 2000, 2007, 2010, 2011 and 2016 are considered as La Niña years in the present analysis. ONI data set used in the present study is available through https://origin.cpc.ncep.noaa.gov.

### Pacific Decadal Oscillation

In order to examine the effect of PDO on the observed trends in CTH over the NWIO, PDO Index data provided by the Joint Institute for the Study of the Atmosphere and Ocean (JISAO) through http://research.jisao.washington.edu/pdo/ are used^57^. JISAO provides PDO index using UKMO Historical SST for the period 1900–1981, Optimum Interpolation SST (OISST) Version1 for 1982–2001 and OISST Version 2 from 2002 onwards. PDO Index shows both positive and negative values during the study period. Warm and cold phases of PDO during the study period are identified by considering the years of positive and negative values of PDO Index. Trends in CTH over the NWIO are estimated separately for the warm and cold phases of PDO.

### TOA fluxes and CRE from CERES

Changes in TOA fluxes and CRE are examined using the Energy Balanced and Filled (EBAF Ed4.1) level 3b data from the Clouds and the Earth’s Radiant Energy System (CERES)^58^. This data set is available through https://ceres.larc.nasa.gov. The data set includes TOA outgoing shortwave and long wave radiations under clear sky and all sky conditions, which are used to compute CRE_SW_, CRE_LW_ and CRE_NET_. CRE_SW_ is computed as, CRE_SW_ = F_SW Clear Sky_ − F_SW All Sky_, where F_SW Clear Sky_ and F_SW All Sky_ are the TOA shortwave fluxes under clear sky and all sky conditions respectively. Similarly, CRE_LW_ is computed as CRE_LW_ = F_LW Clear Sky_ − F_LW All Sky_, where F_LW Clear Sky_ is the TOA longwave flux under clear sky conditions and F_LW All Sky_ is the same under all sky conditions. Net cloud radiative effect is obtained as the resultant of shortwave and longwave cloud radiative effects (CRE_NET_ = CRE_SW_ + CRE_LW_). Trends of the TOA outgoing fluxes and CREs are estimated, by excluding the years of El Niño and La Niña conditions. Estimated annual trends are multiplied with the total number of years to obtain the changes in fluxes and CREs during the study period.

## Supplementary Information

Supplementary Information.
