# Supplementary material for: Intra-seasonal contrasting trends in clouds due to warming induced circulation changes
Source: Sci Rep. 2021 Aug 20;11:16985. doi: 10.1038/s41598-021-96246-2 (PMC8379180; doi:10.1038/s41598-021-96246-2)
Supplement: Supplementary file 1 — Supplementary Information. [file 41598_2021_96246_MOESM1_ESM.docx]

Supplementary Material

**Intra-seasonal contrasting trends in clouds due to warming induced circulation changes**

***S. S. Prijith, C. B. Lima, M. V. Ramana and M. V. R. Sesha Sai**

# National Remote Sensing Centre

# Indian Space Research Organisation

# Hyderabad, India

# *Corresponding author: Email: prijithss@gmail.com

Phone No: +91 8542225169 Fax No: +91 40 23875932


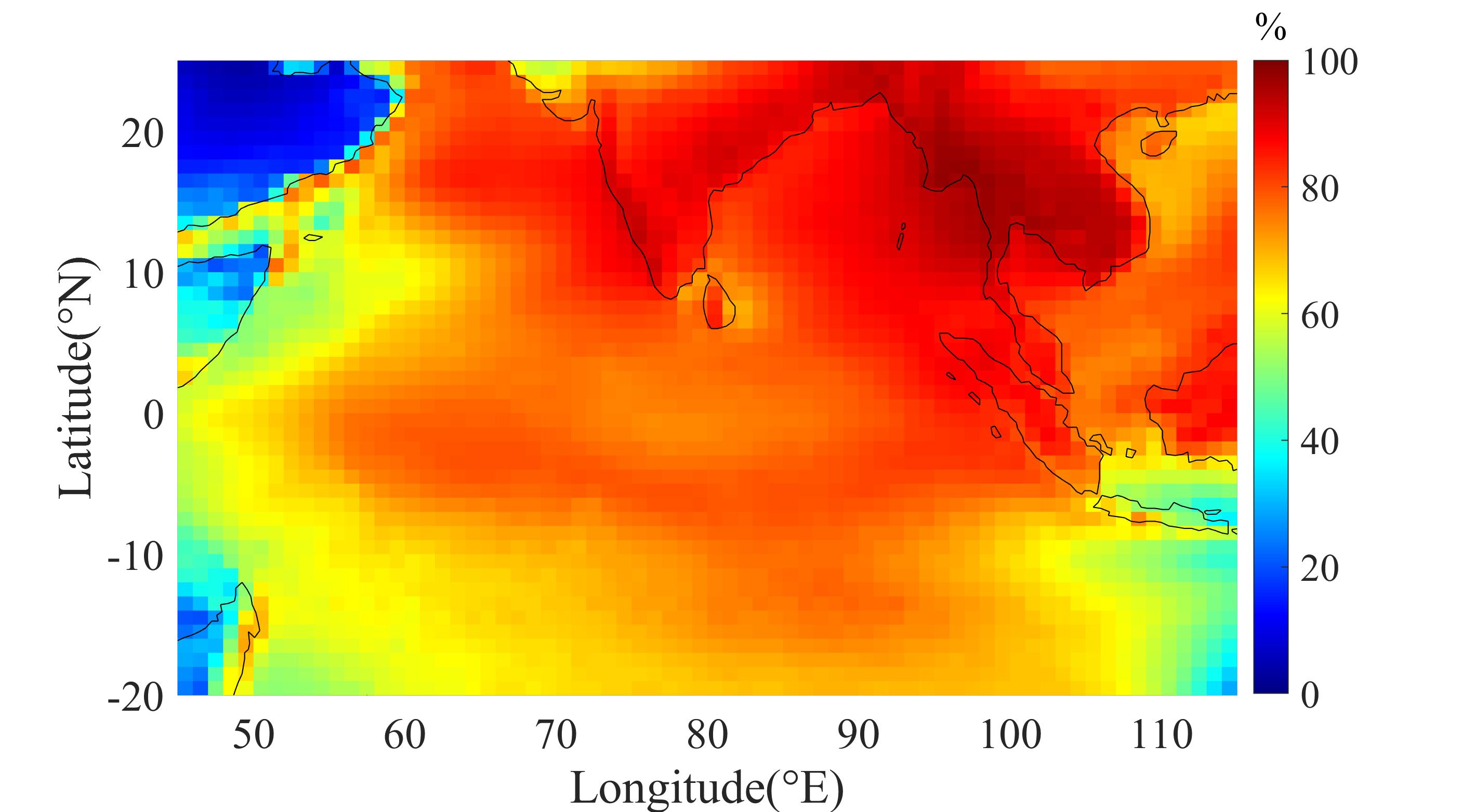

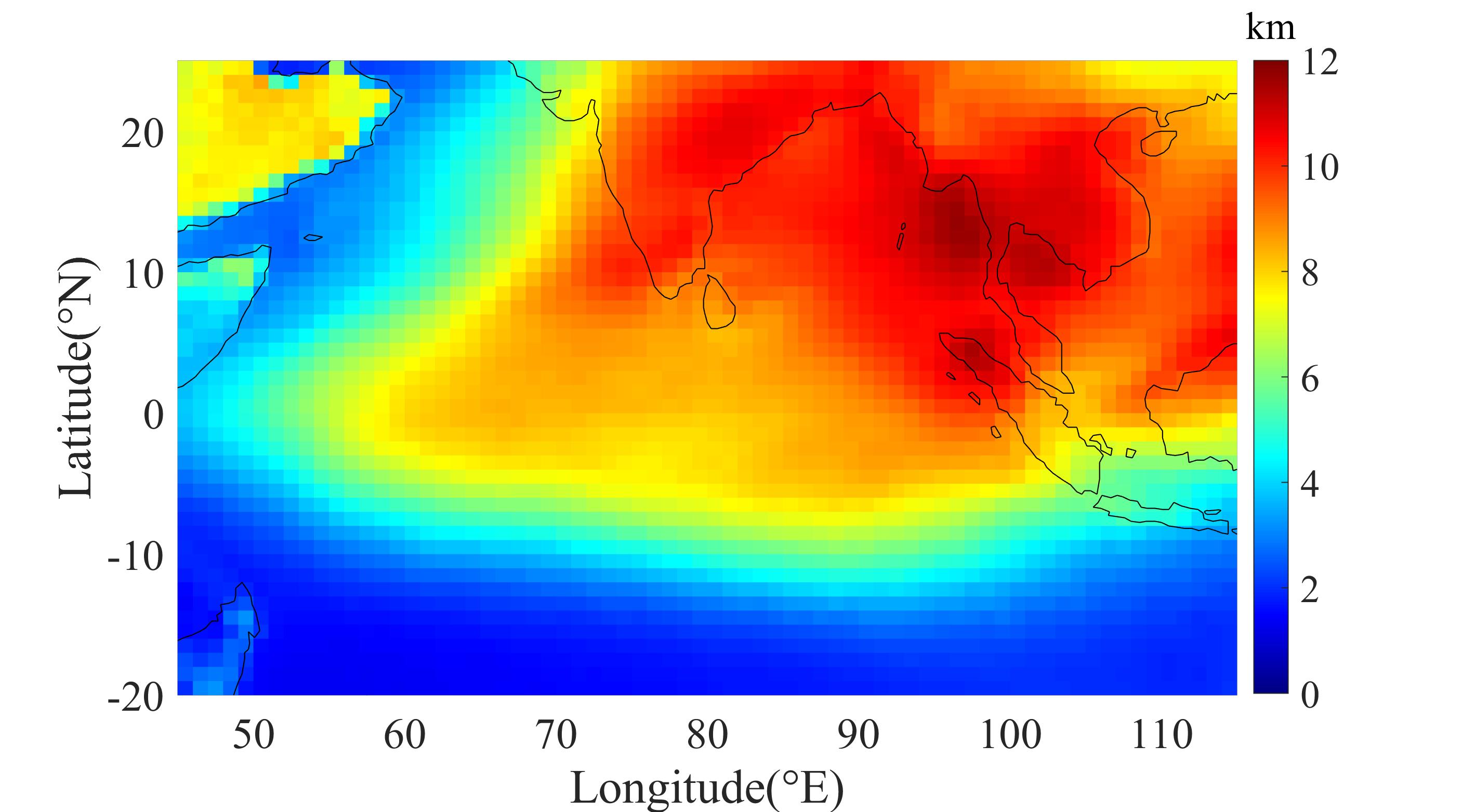


Mean (a) cloud fraction (CF) and (b) cloud top height (CTH) during summer monsoon (June to September), from MODIS-Terra measurements for the period, 2000 to 2017. Both CF and CTH are observed to be higher over the eastern parts of North Indian Ocean (NIO), compared to those over the western parts. This shows large cloudiness at higher altitudes over the Northeast Indian Ocean during the summer monsoon. The map is generated using MATLAB 2020a, www.mathworks.com.

(a)

(b)

**Fig. S1. Spatial pattern of cloudiness over NIO in summer monsoon**

**Fig. S2: Spatial variations in trends of CF in summer monsoon months**

Spatial variations of trends in CF from MODIS-Terra during 2000 to 2017, in the months of (a) June, (b) July, (c) August and (d) September. Positive values indicate the regions, where CF enhances and negative values represent the regions, where CF diminishes. Black dots show the regions where the trends are not statistically significant at 95% confidence level. The map is generated using MATLAB 2020a, www.mathworks.com.


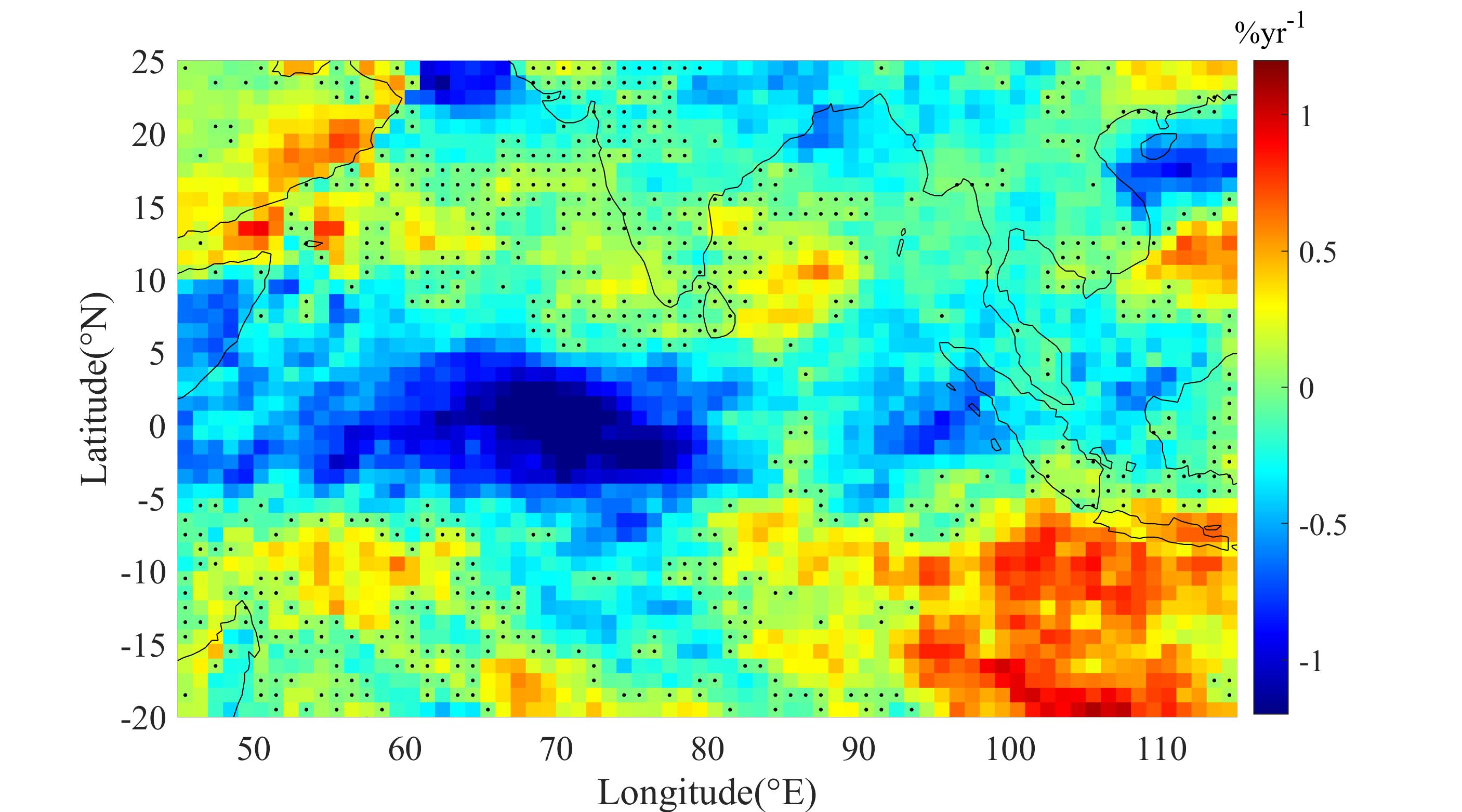

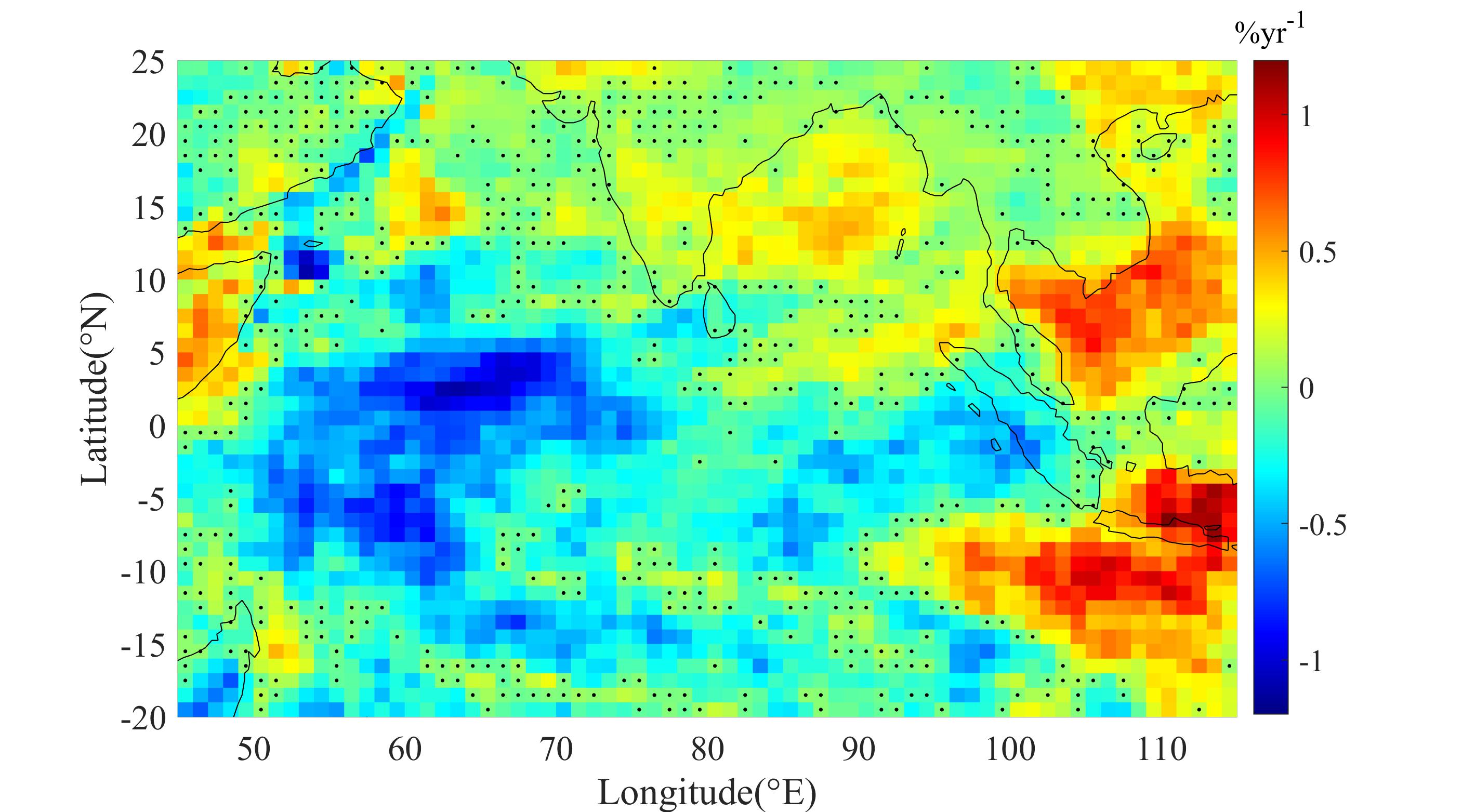


(a)

(b)


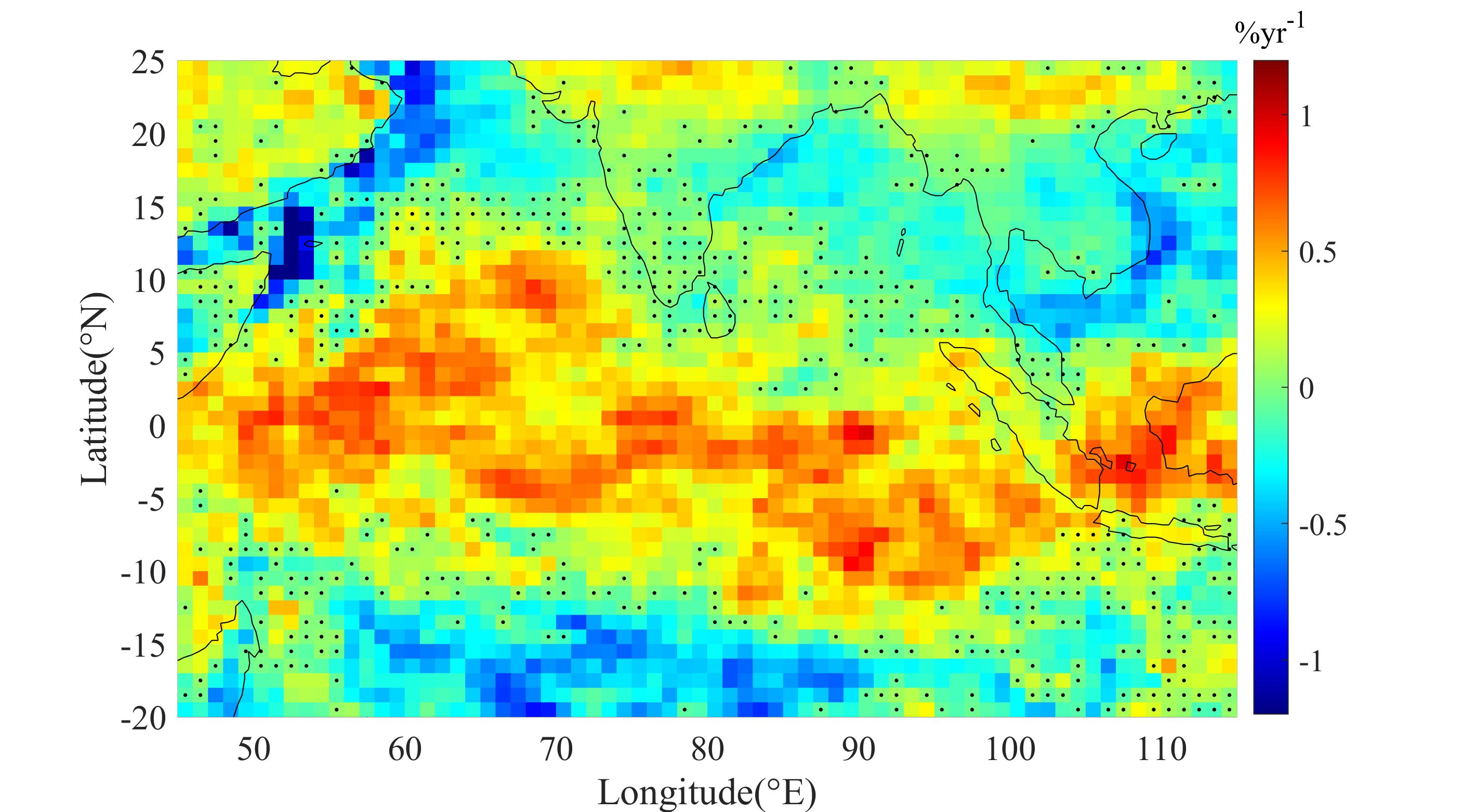

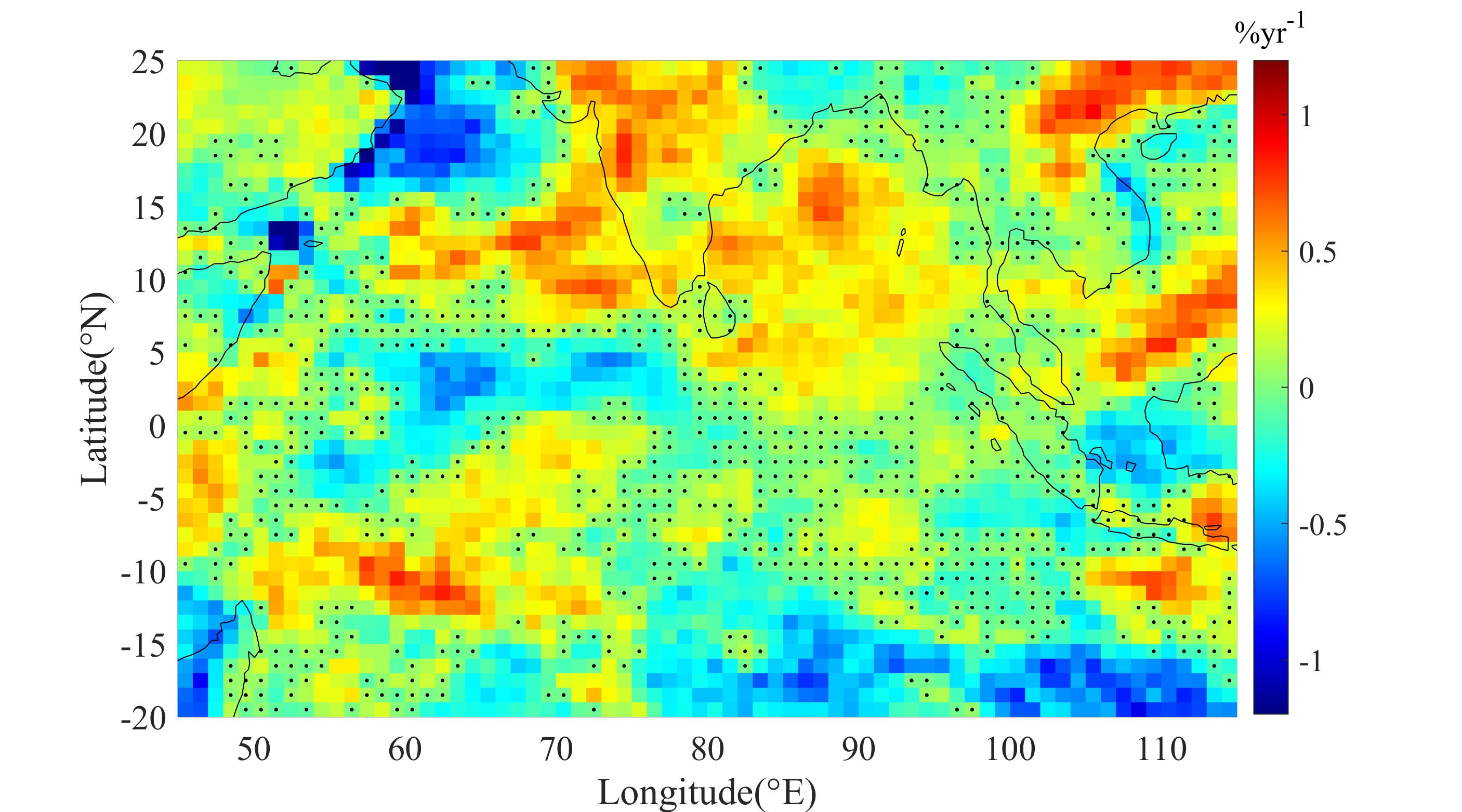


(d)

(c)

Spatial variations of trends in Cloud Top Height (CTH) in (a) June and (b) August, from MODIS-Terra measurements during 2000 to 2017, by excluding El Niño and La Niña years. Positive values show the regions, where CTH is increasing and negative values indicate those where CTH is decreasing. Black dots show the regions where the trends are not statistically significant at 95% confidence level. The map is generated using MATLAB 2020a, www.mathworks.com.


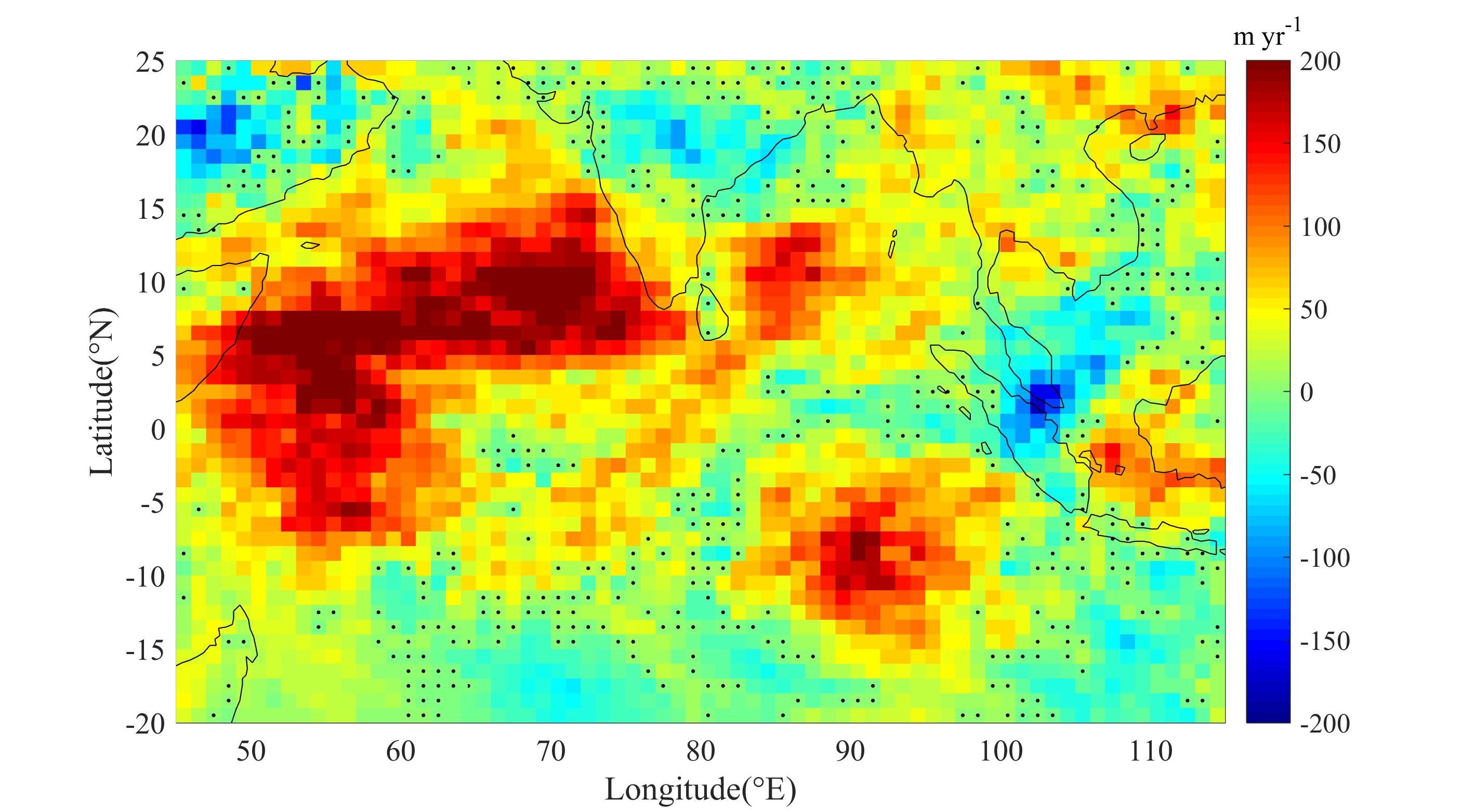


(b)

**Fig. S3: Spatial variations in trends of CTH over equatorial Indian Ocean**


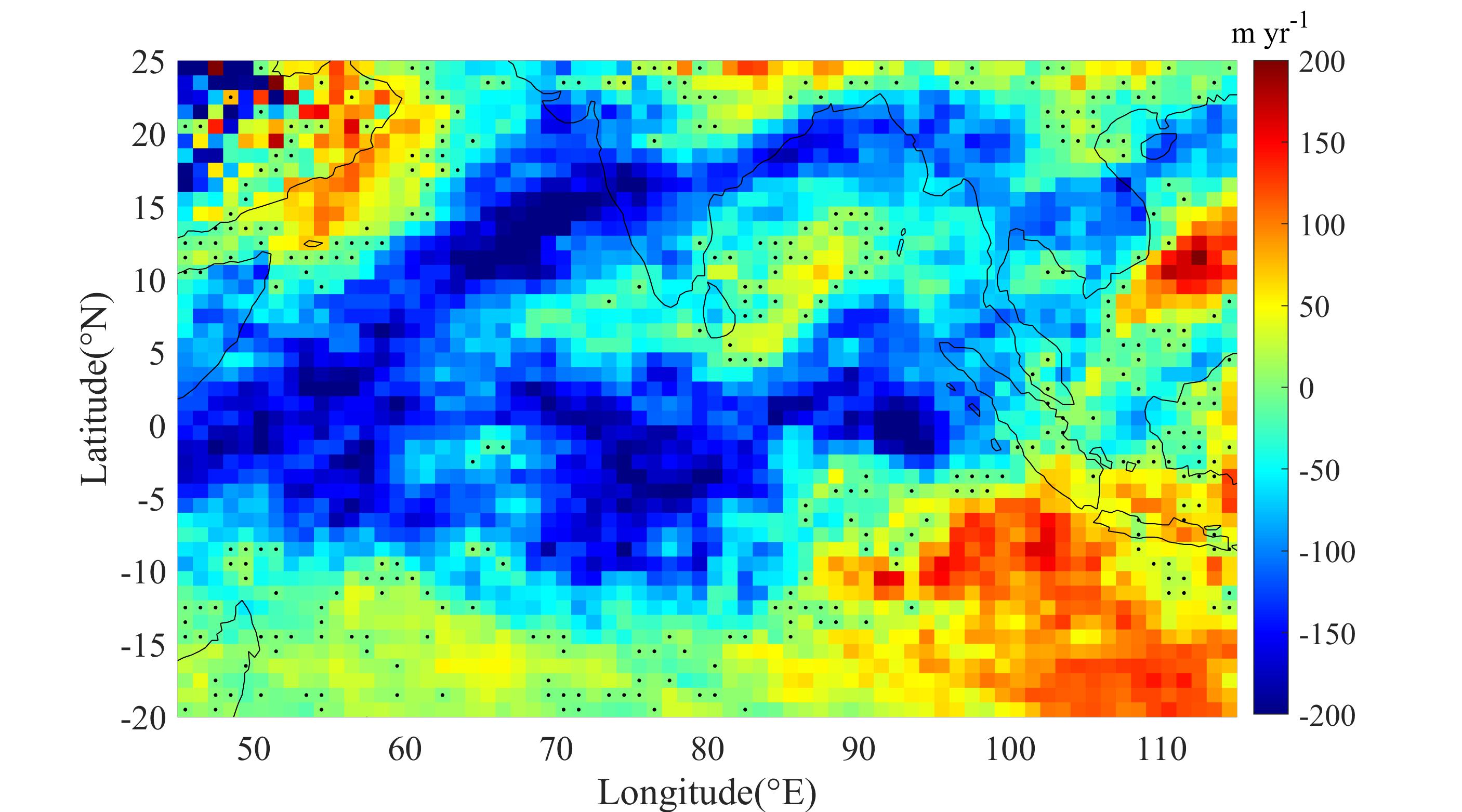


(a)


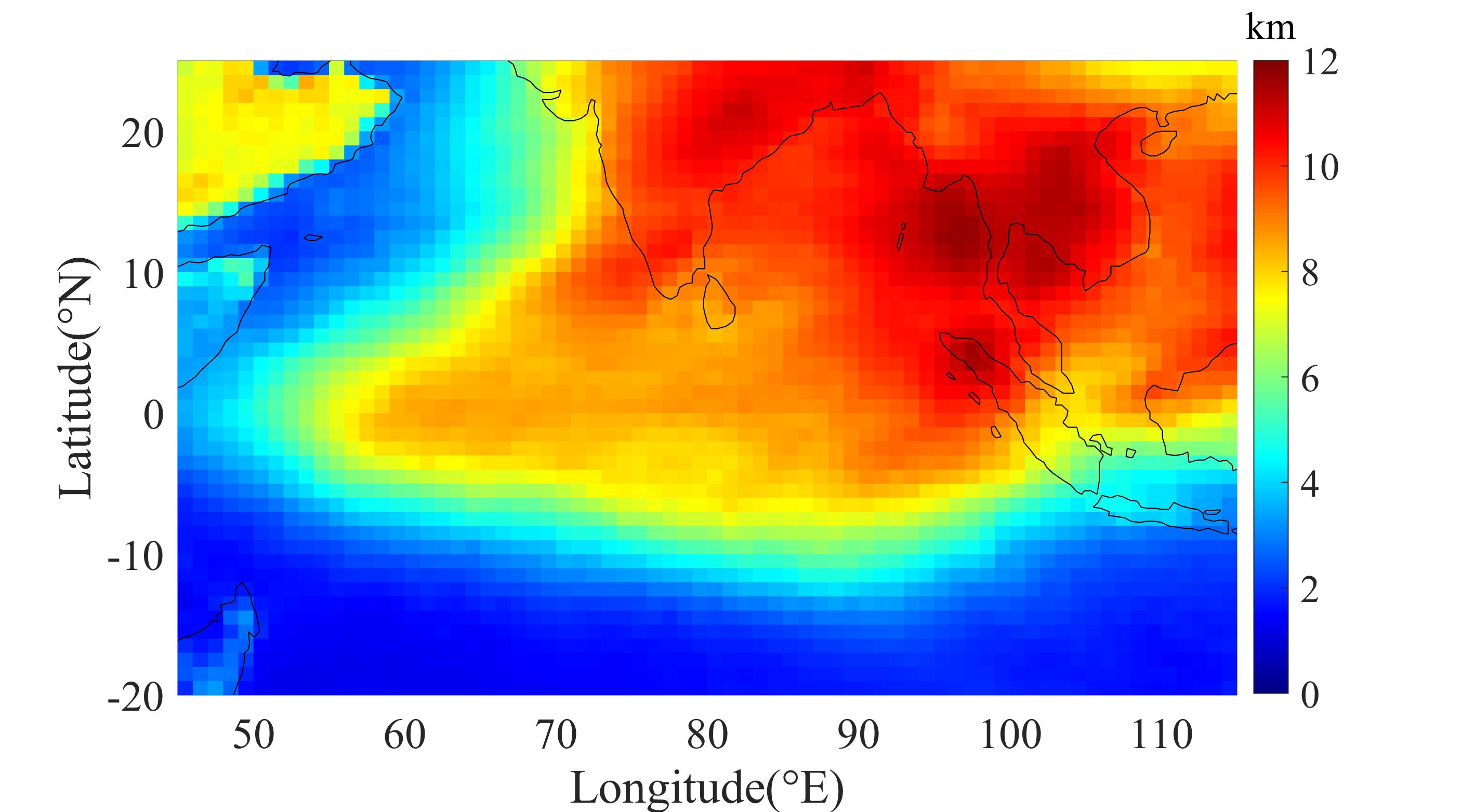

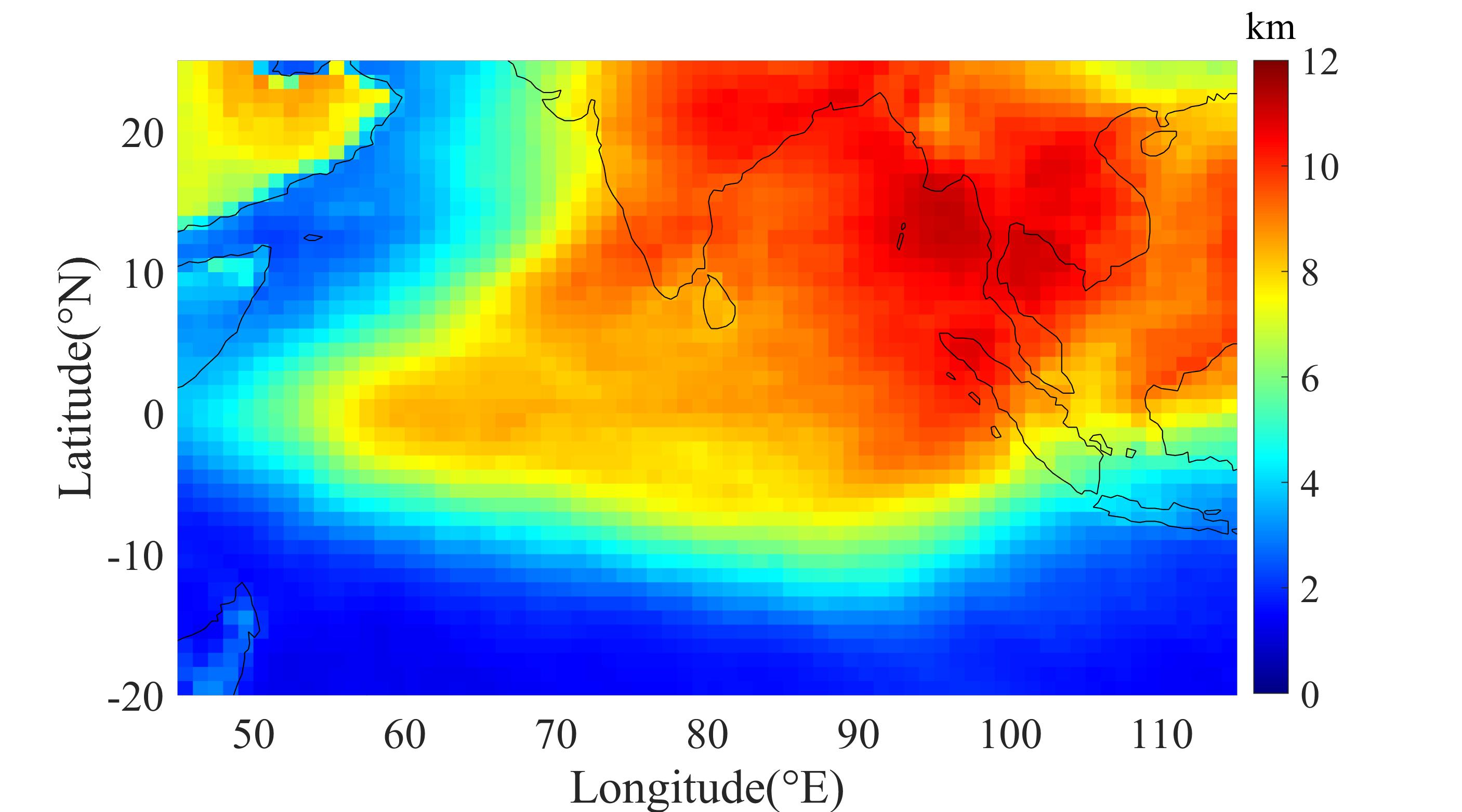


Spatial pattern of CTH in the month of August from (a) MODIS-Terra observations from 2000 to 2017 and (b) MODIS-Aqua observations from 2002 to 2017. CTH is higher over the eastern regions of NIO, compared to its western parts, indicating the clouds at higher altitudes (>7km) over NEIO. The map is generated using MATLAB 2020a, www.mathworks.com.

**Fig. S4. Spatial pattern of CTH in August over NIO**

(a)

(b)

Cloud Fraction (CF) in the month of August, averaged over the latitude band between 5°S and 15°N, from MODIS onboard Terra. The figure shows westward spread of clouds over north Indian Ocean during the period from 2000 to 2017, as indicated by the black arrow.

**Fig. S5: Westward spread of clouds**


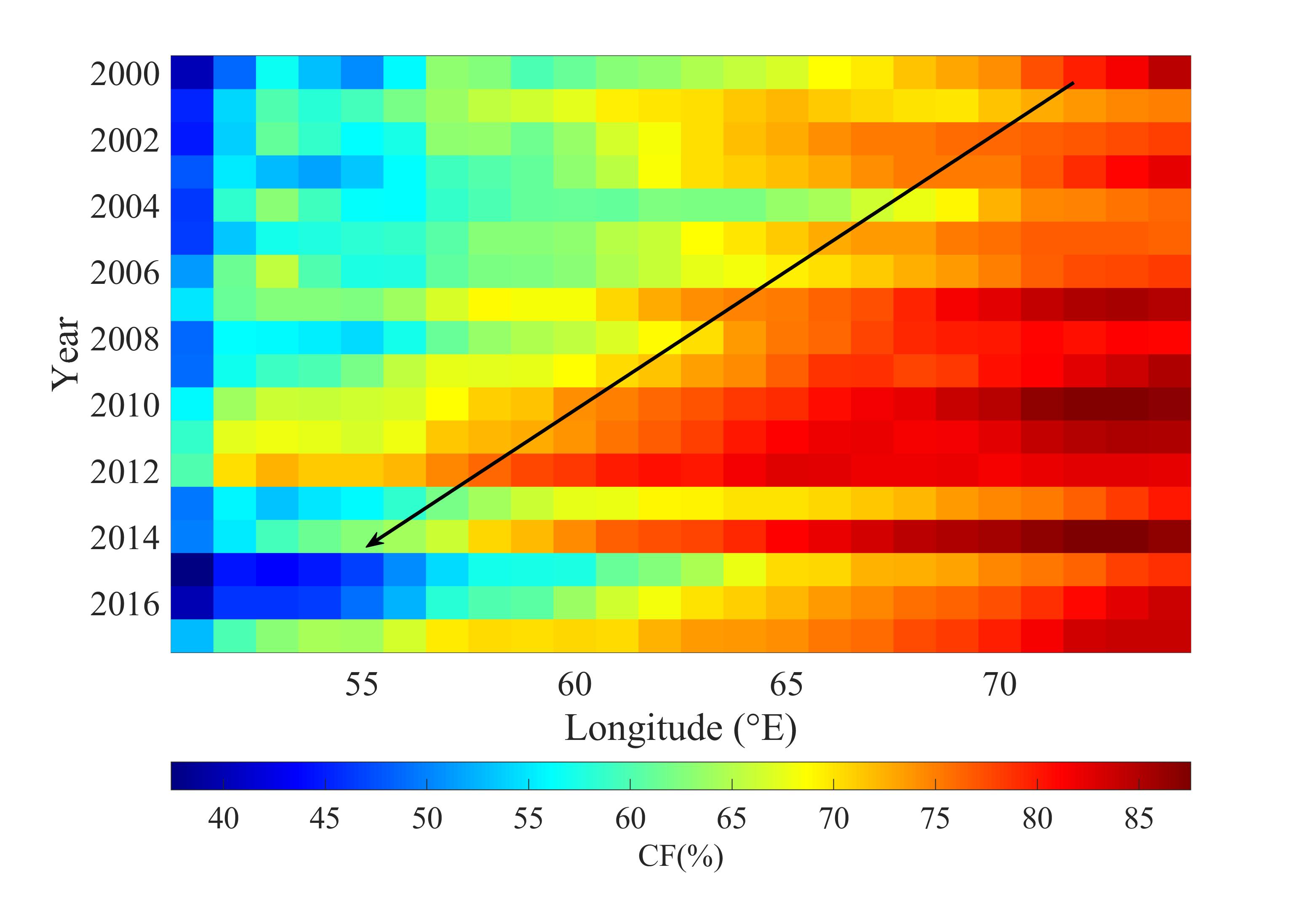


Westward shift of LON_7km_, from MODIS-Terra during 2000 to 2017 and MODIS-Aqua during 2000 to 2017. LON_7km_ is the mean longitude, averaged over the latitude band between 5°S and 15°N, with clouds at east of it above 7km and those at west of it at lower altitudes. Decrease of LON_7km_ during the period shows the westward drift of high level clouds.


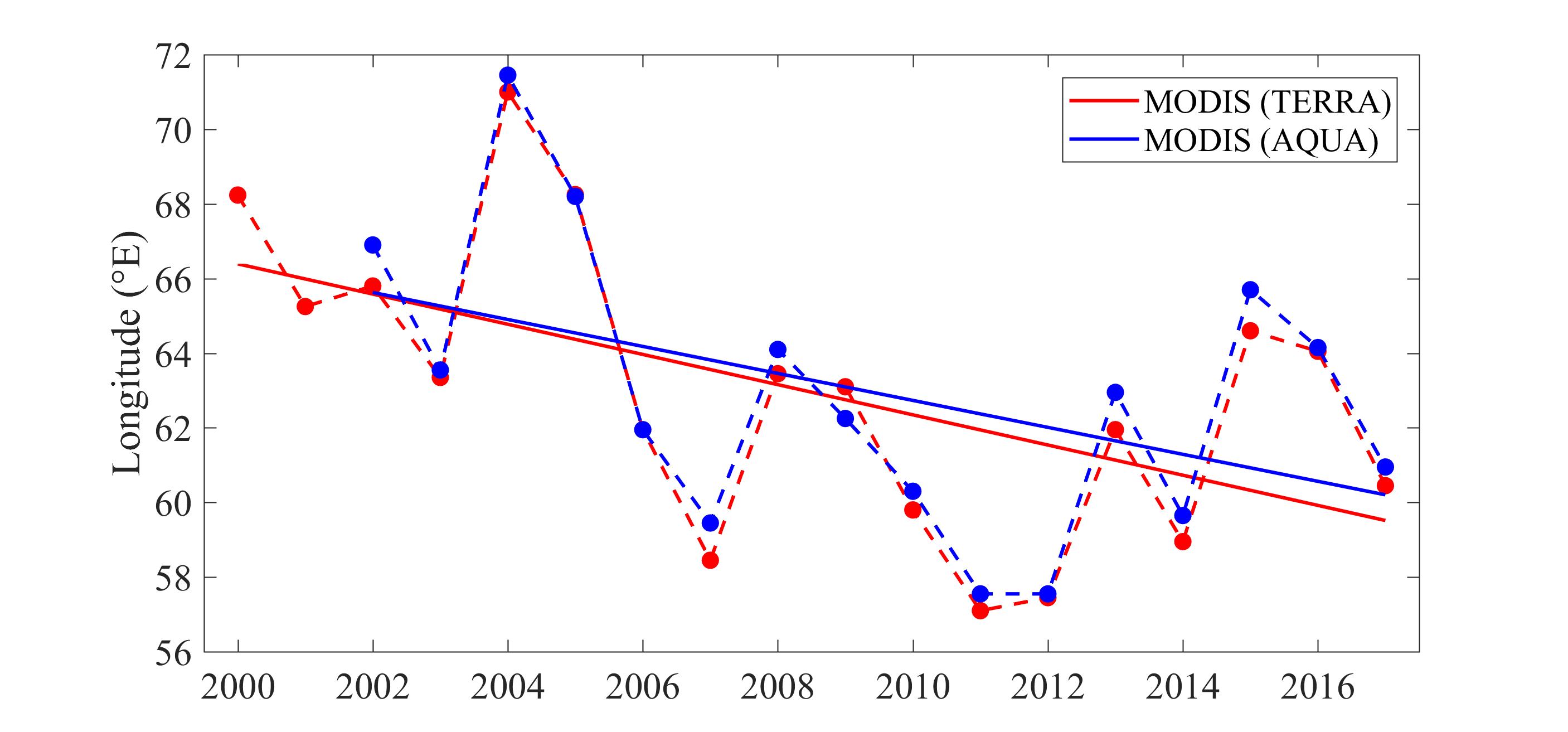


**Fig. S6: Westward shift of LON_7km_**

Colors indicate trends of wind speed and arrows show mean wind pattern at 200hPa in (a) June, (b) July and (c) August during 2000 to 2017. White patches show the regions where the trends are not statistically significant at 95% confidence level. Positive values show the regions, where the winds are strengthening and negative values show those where the winds are weakening. The map is generated using MATLAB 2020a, www.mathworks.com.


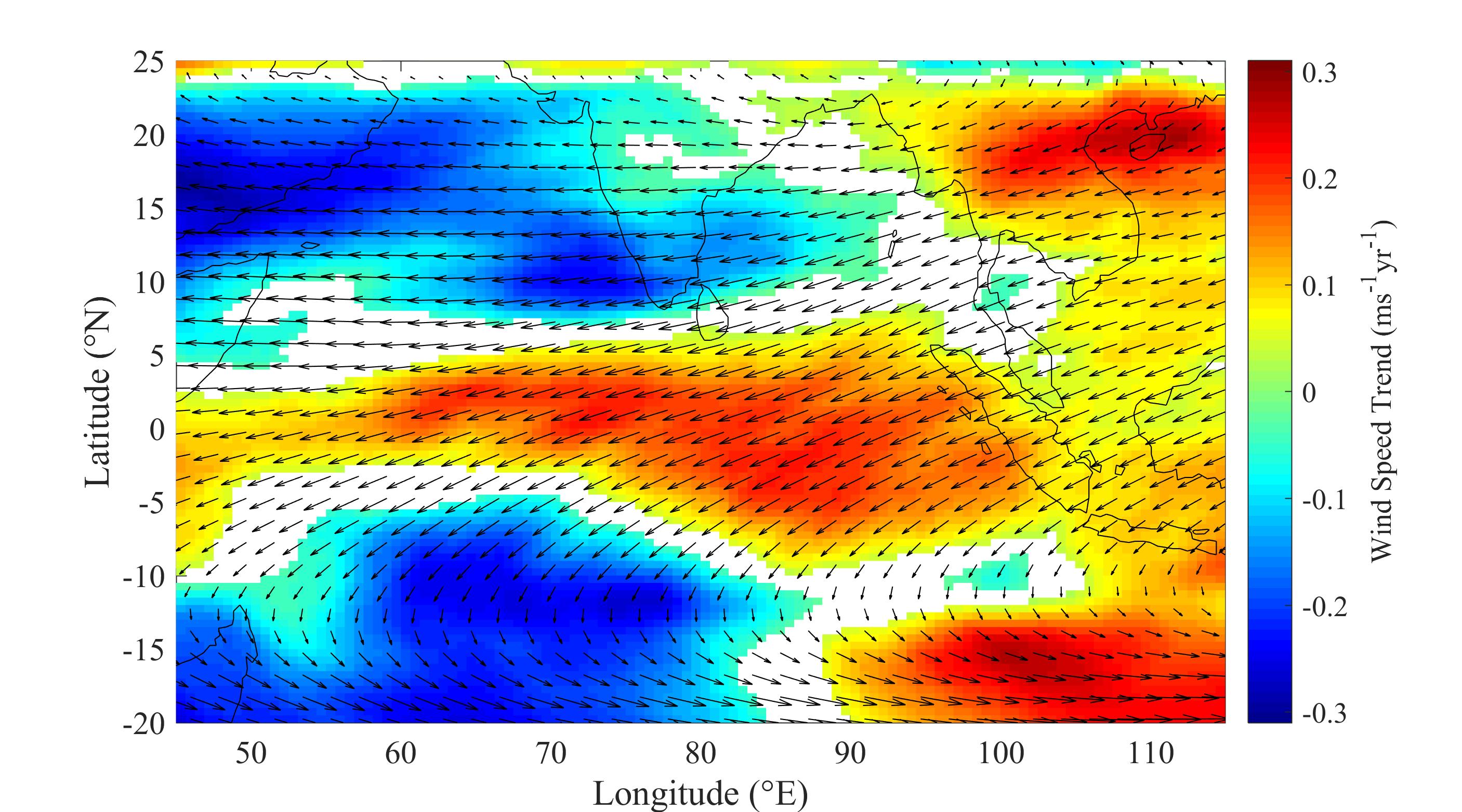

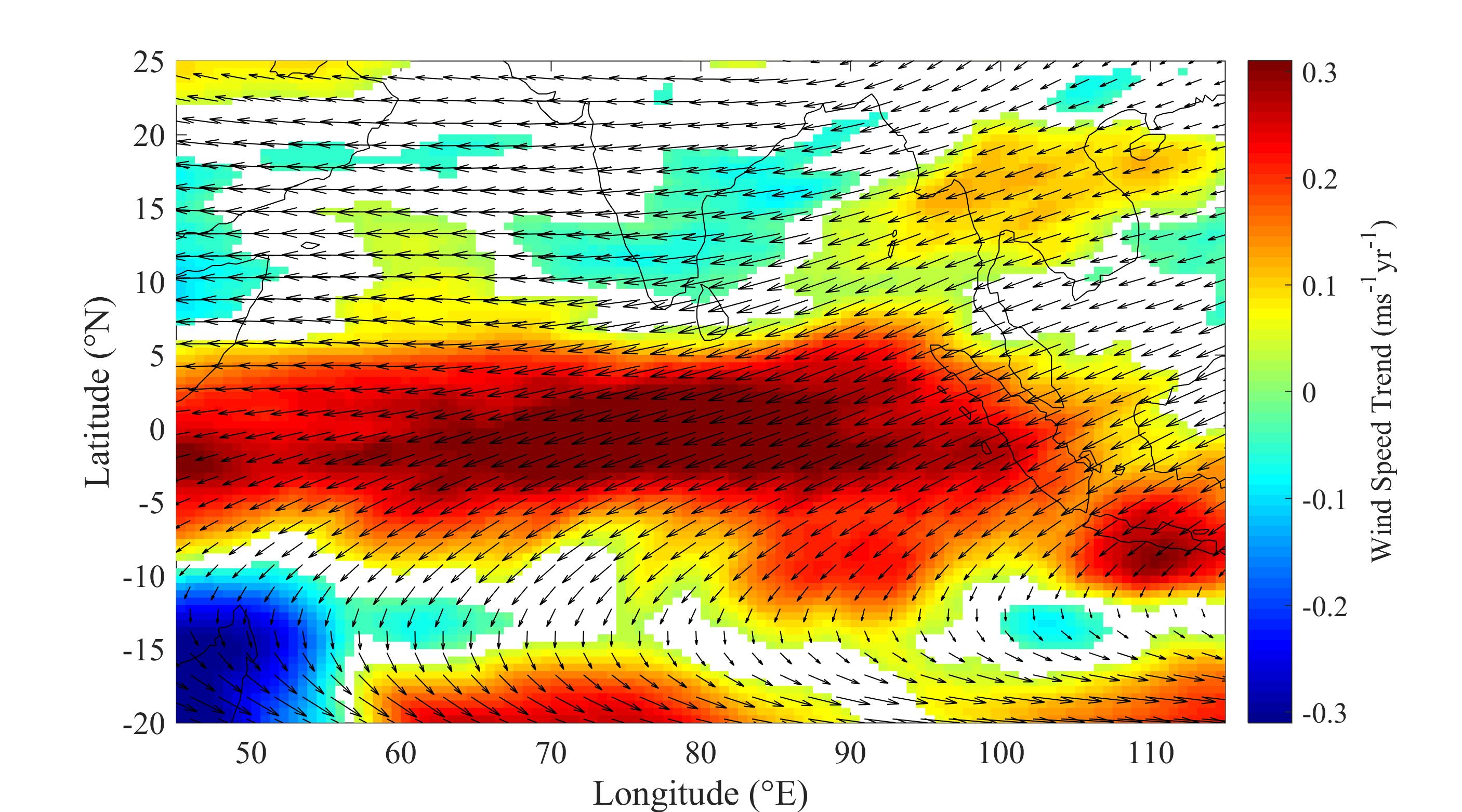

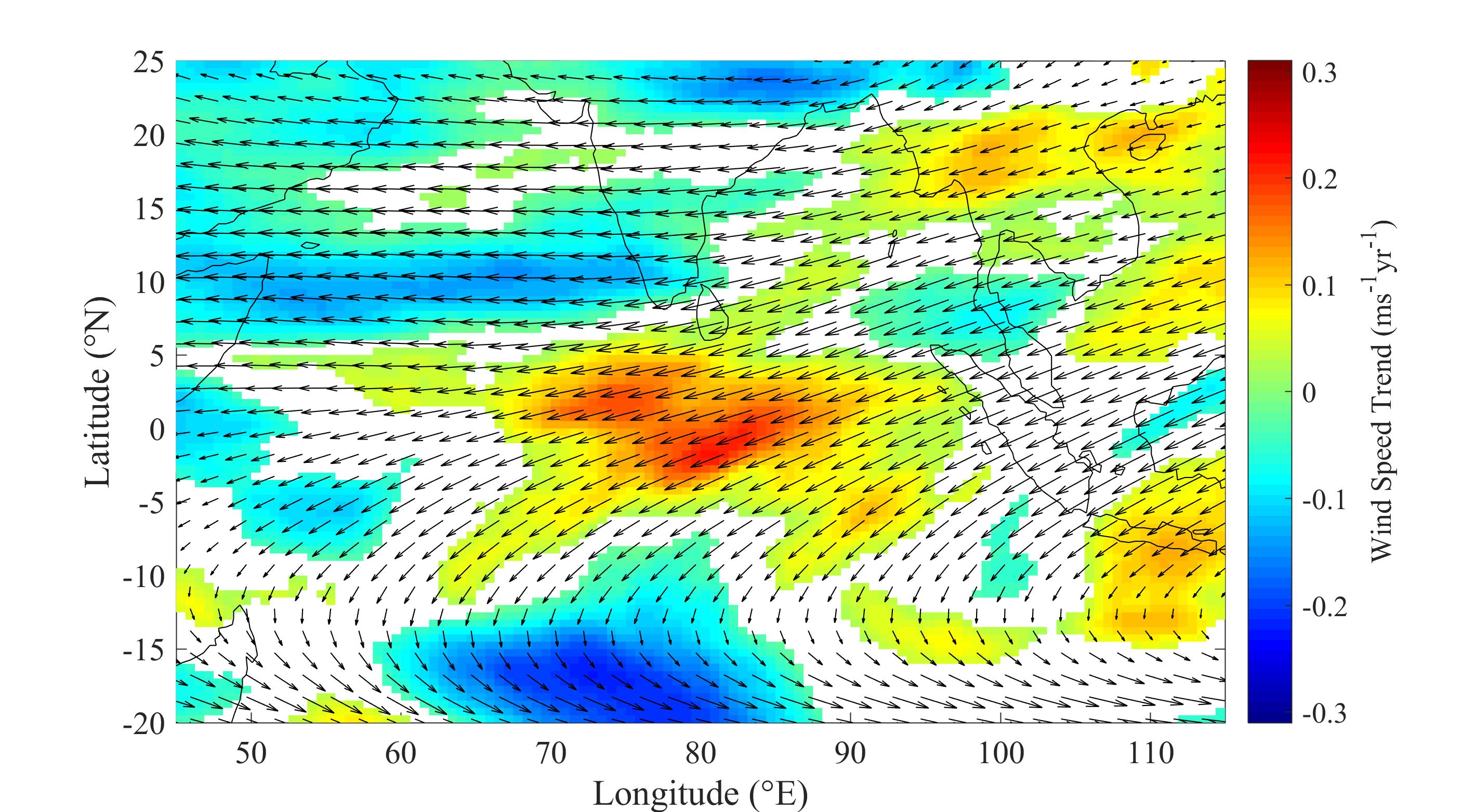


(b)

(c)

(a)

**Fig. S7: Trends of upper tropospheric winds**

Longitude-altitude cross section of mean vertical wind (in Pas^-1^), averaged over the latitude band between 10°S and equator, in (a) June, (b) July and (c) August during 2000 to 2017, by excluding the years of El Niño and La Niña conditions. Positive values (indicated by the colors) show regions of downdraft and negative values represent those of updraft. Black arrows embedded in the figure are generated by considering zonal wind (in ms^-1^) and vertical wind (in -100×Pa s^-1^), only to indicate the wind pattern.


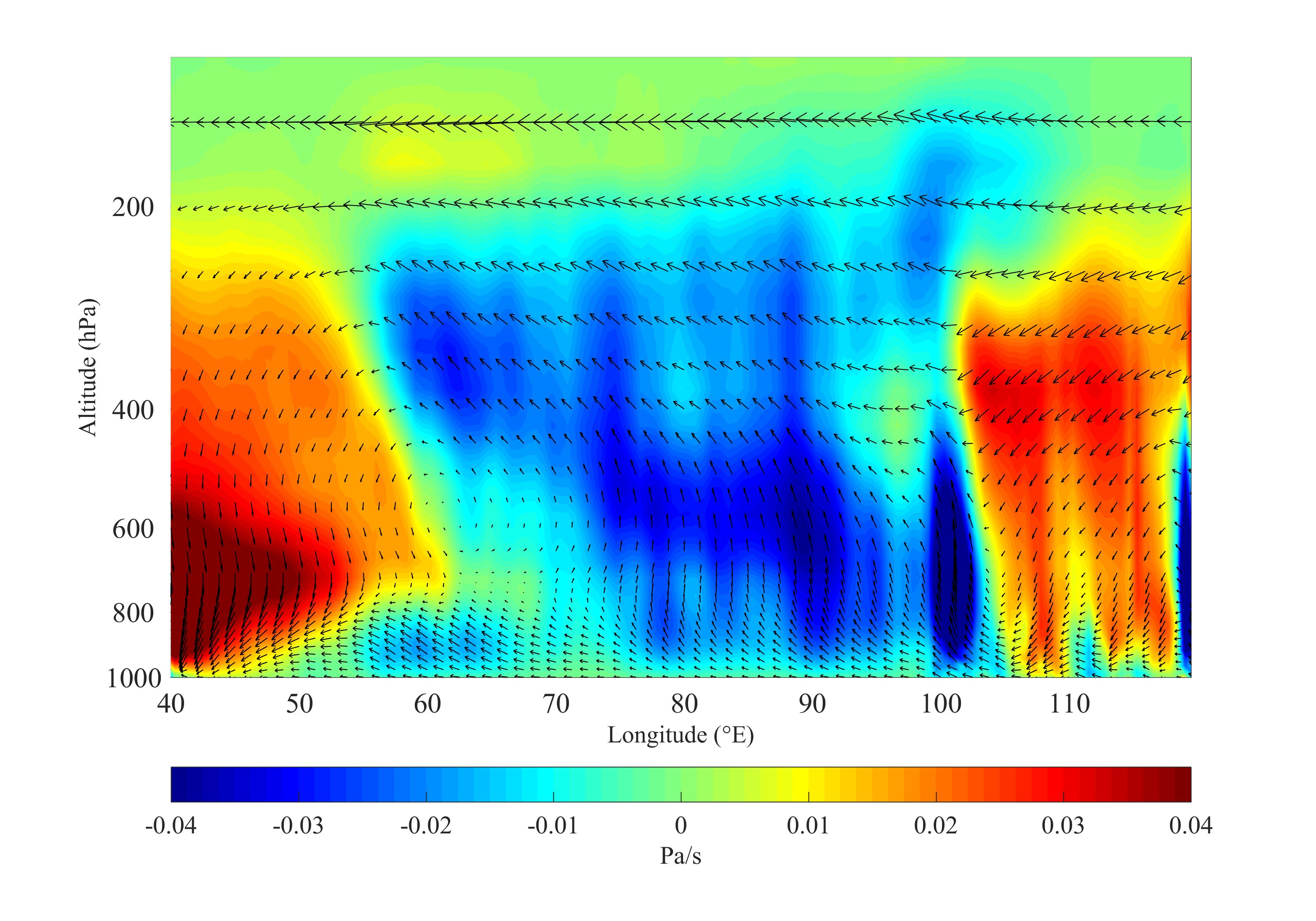


**Fig. S8: Mean vertical wind and Indian Ocean Walker Cell**


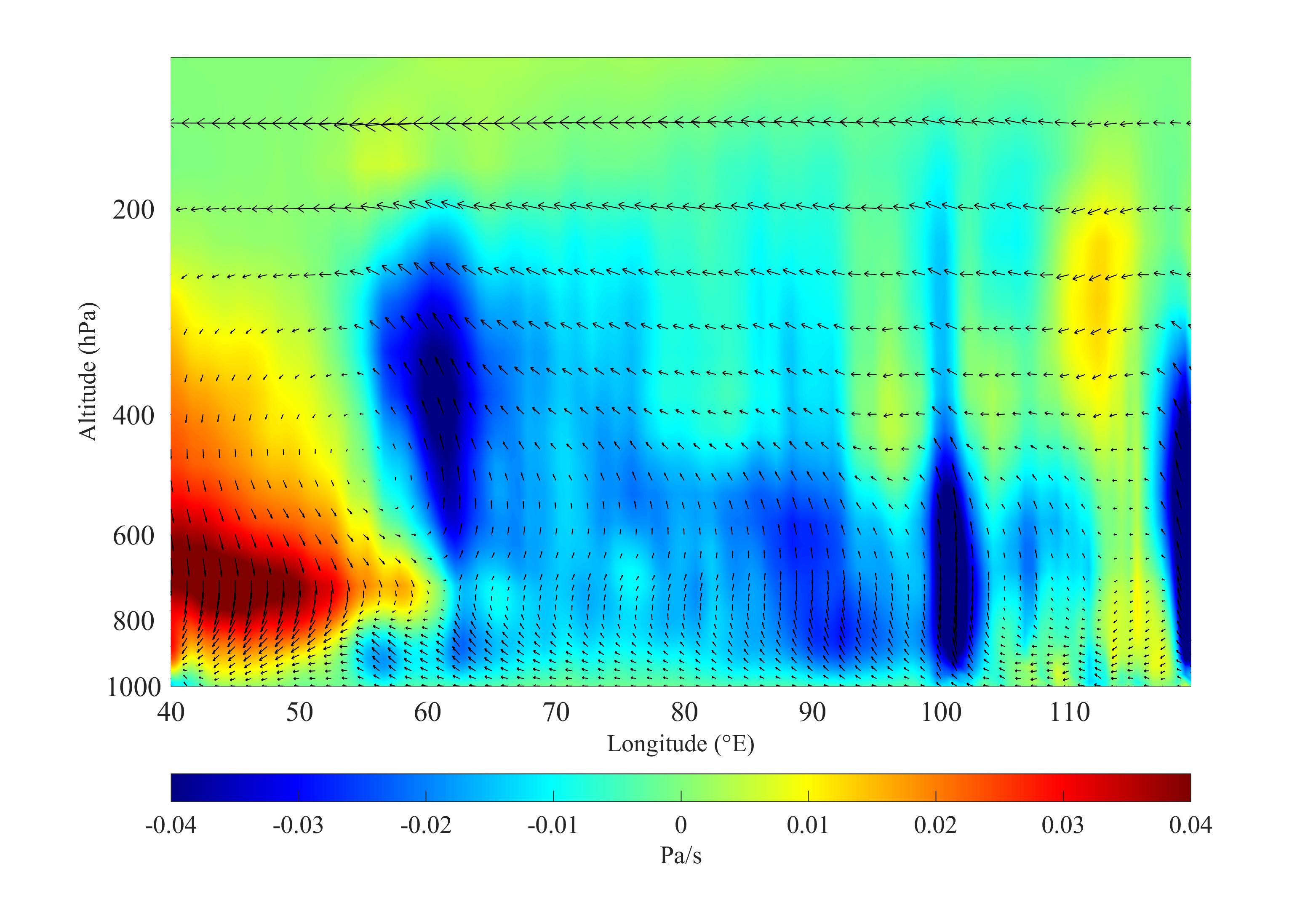

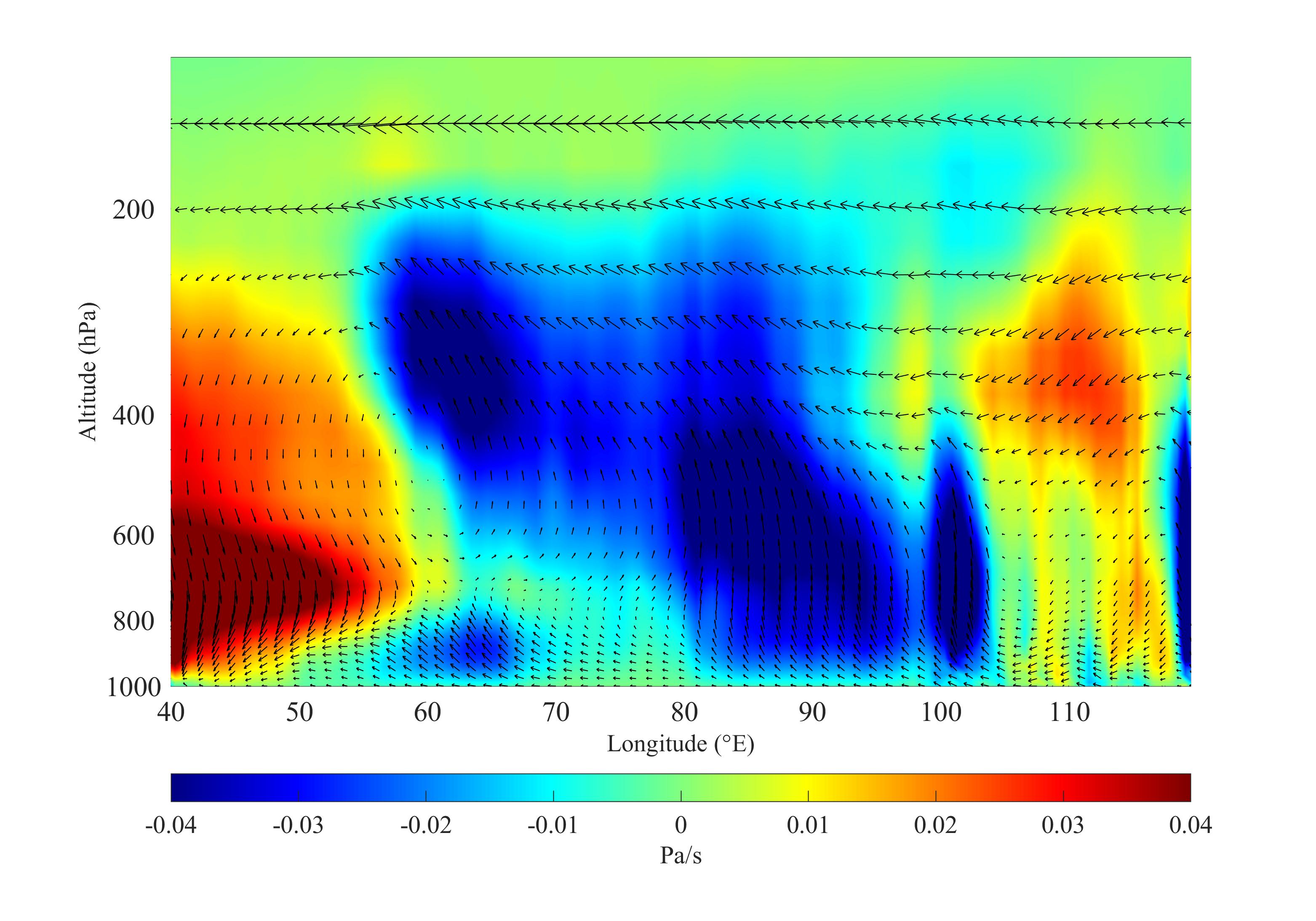


(a)

(b)

(c)

Difference in mean vertical wind at the altitude levels from 600hPa to 400hPa, over the regions between 45°E-55°E (West) and 85°E-95°E (East), within the latitude band between 10°S and equator. Filled circles show the mean difference in each year, whereas the solid lines represent the linear trends. while black curves show the inter annual variations and trends during 2000 to 2017, red, green and blue curves show the same by excluding the years of El Niño, La Niña and both of them respectively. Increasing trend of the difference in vertical wind between west and east of north Indian Ocean indicates strengthening of Walker circulation cell over this region.


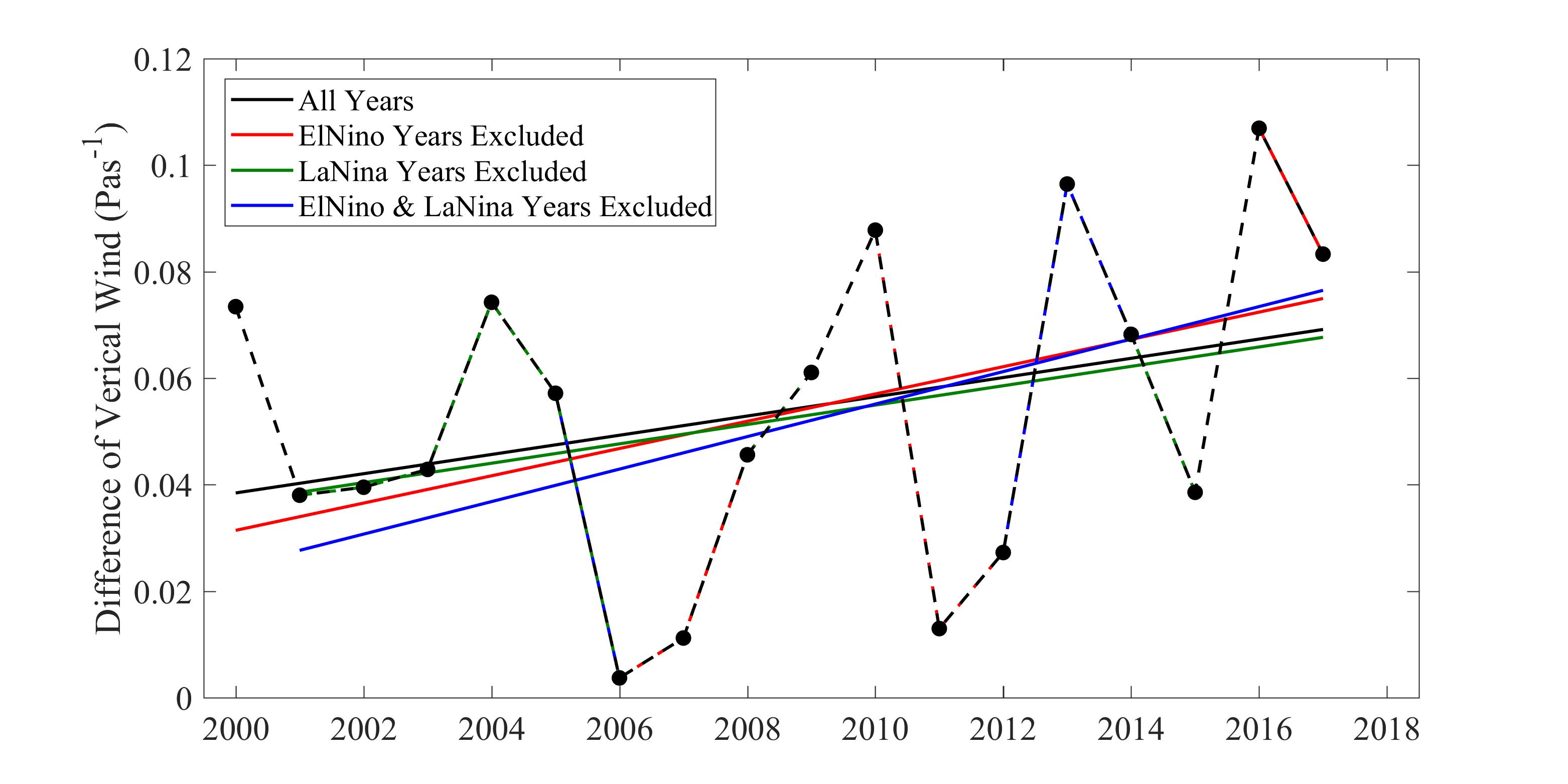


**Fig. S9: Strengthening of Indian Ocean Walker Cell**


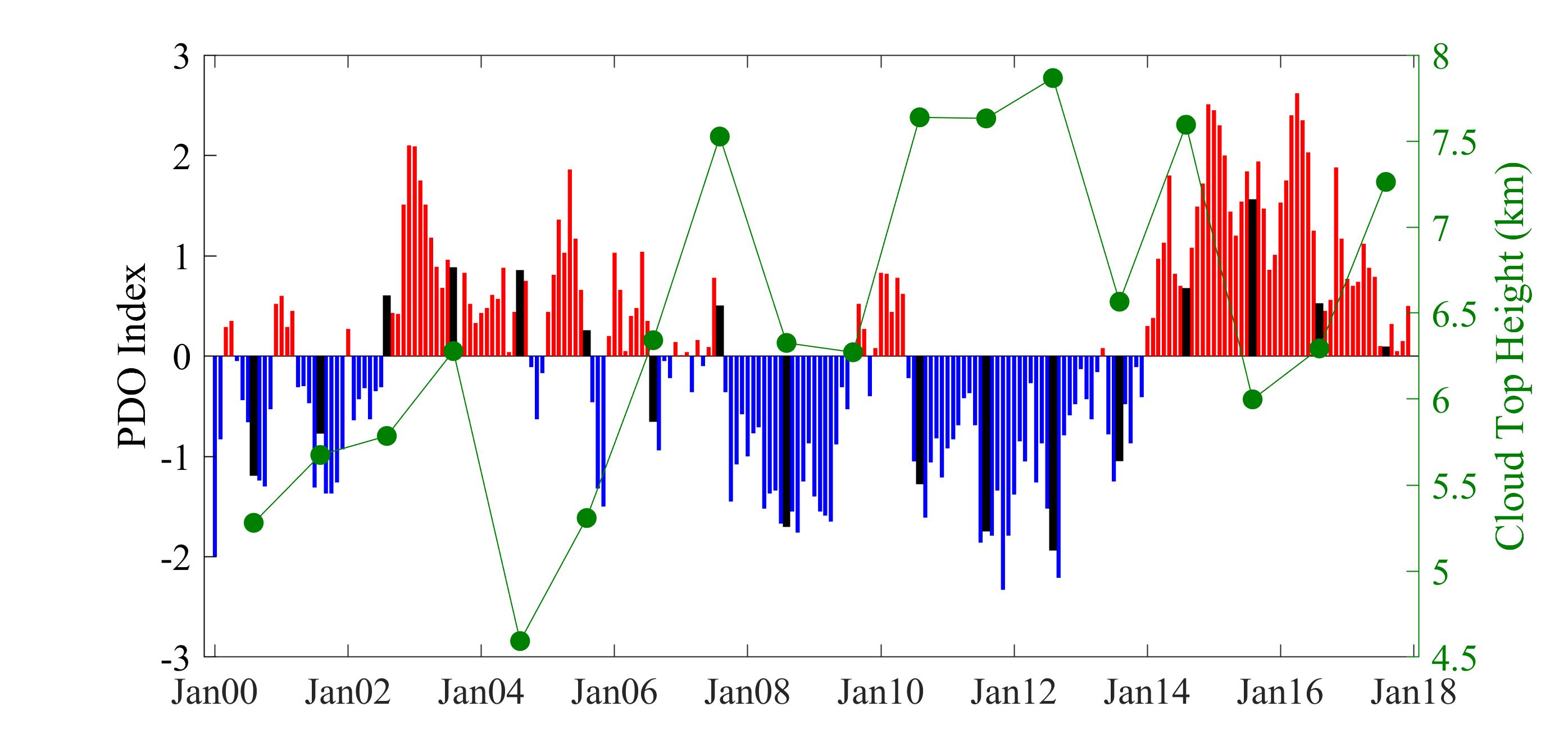


(a) Time series of monthly PDO Index and CTH over NWIO, during the period from January 2000 to December 2017. Red and blue bars show positive and negative values of monthly PDO Index, whereas the black bars represent the PDO Index in the month of August in each year. Green filled circles represent the mean CTH in August over the NWIO. PDO Index values are obtained from http://research.jisao.washington.edu/pdo/.

(b) Time series and trends of CTH in August over the NWIO during the entire study period (black), warm phase of PDO (red) and cold phase of PDO (blue).

**Fig. S10: Effects of PDO on the trends of CTH over NWIO**

(a)


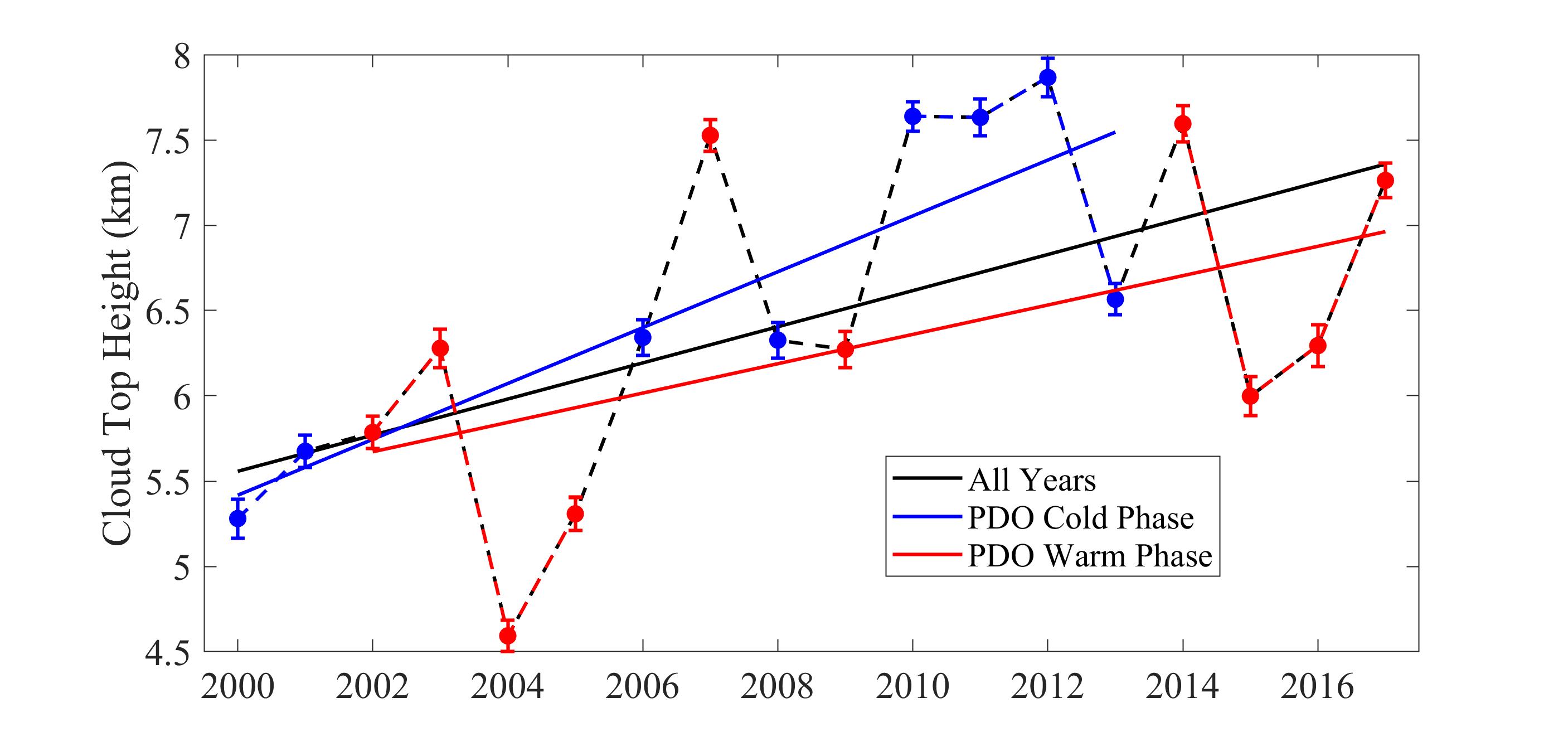


(b)

Inter annual variation of mean CTT and its linear trend over the region 5°S-15°N & 50°E-75°E, in (a) June and (b) August, from MODIS-Terra observations. While filled circles show mean CTT over the region, associated vertical bars represent corresponding standard errors and solid lines indicate the linear trends. Black curves are generated by including the measurements in all the years, whereas red, green and blue are by excluding those in El Niño, La Niña and both El Niño and La Niña years.

**Fig. S11: Intra-seasonal contrasting trends in CTT over NWIO**


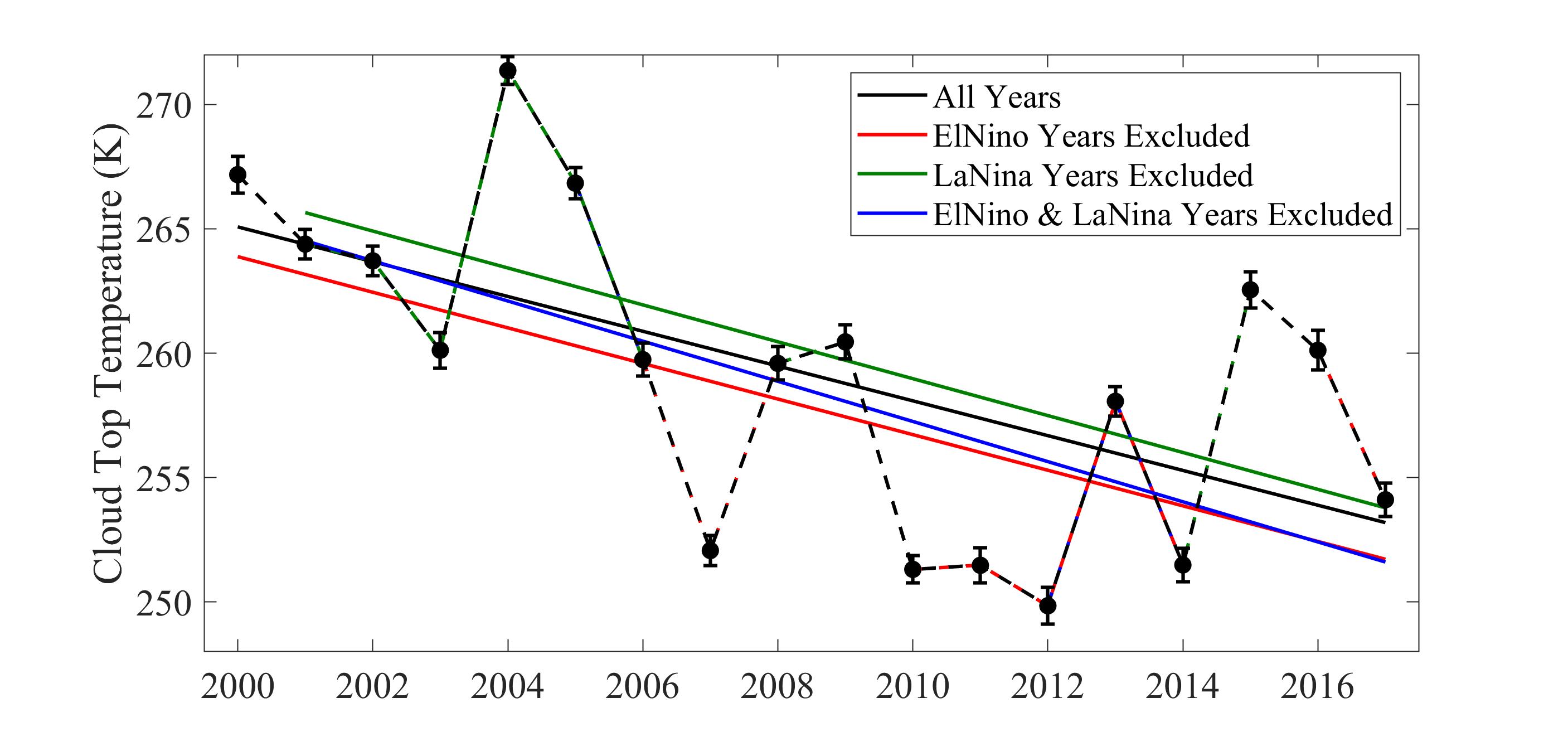


(b)


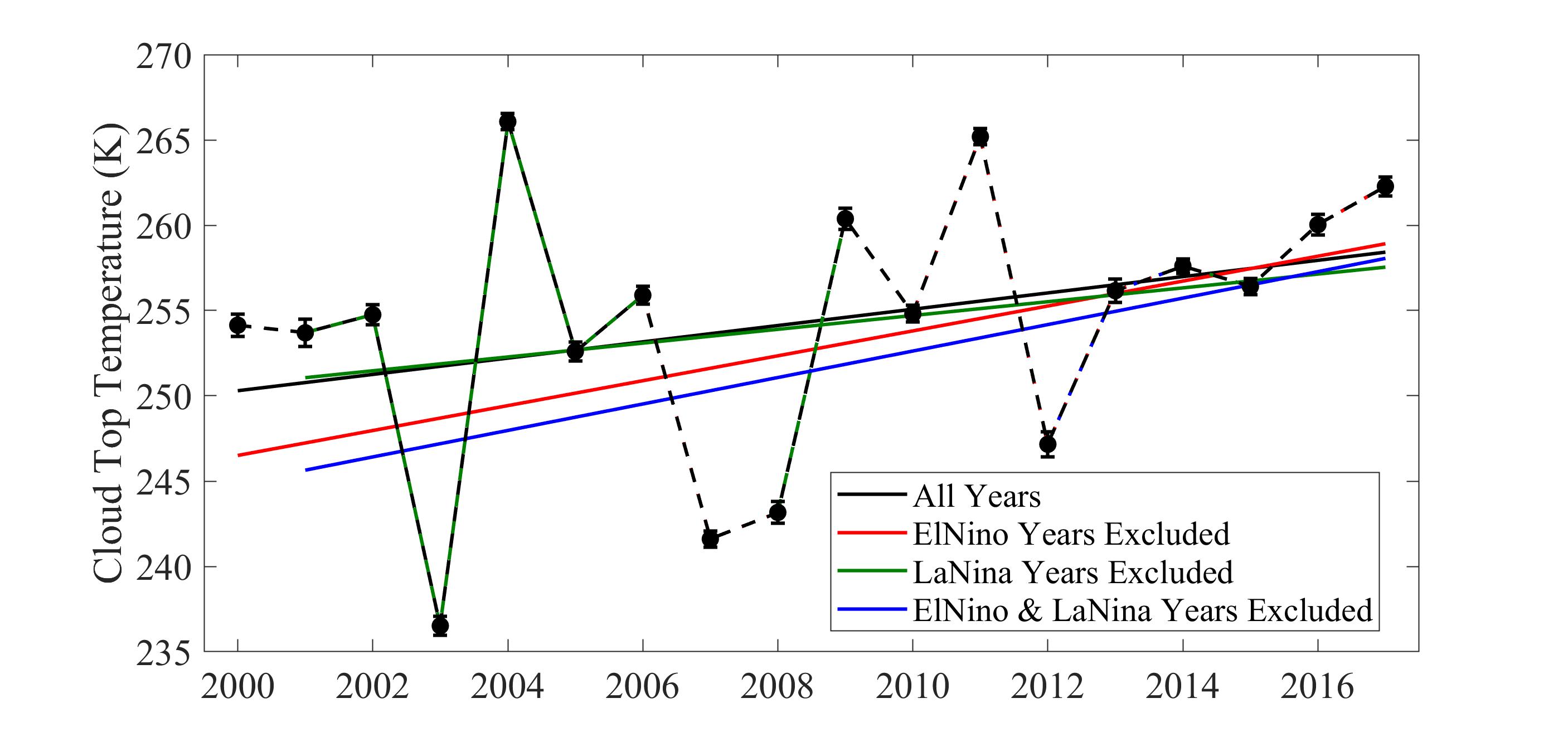


(a)


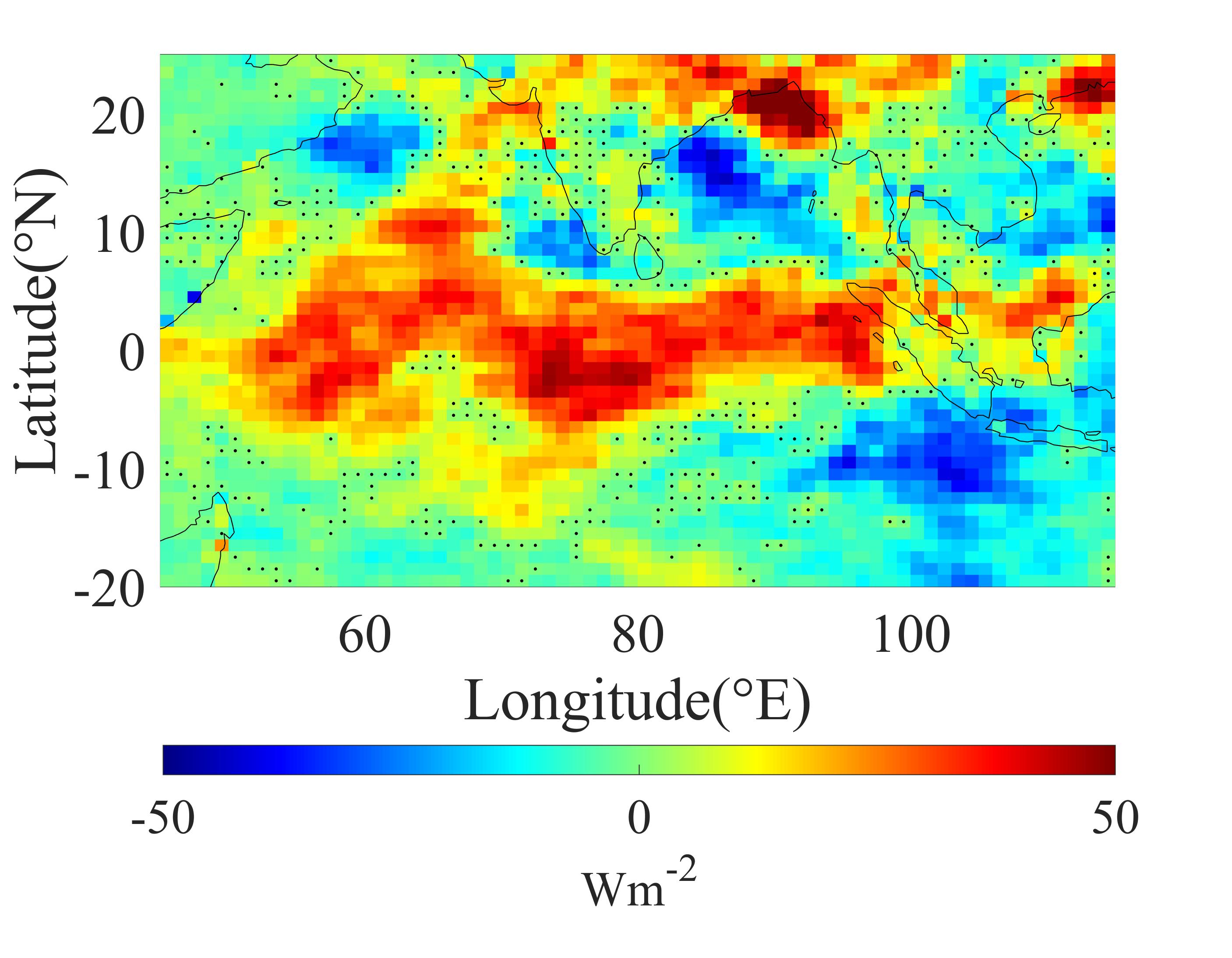

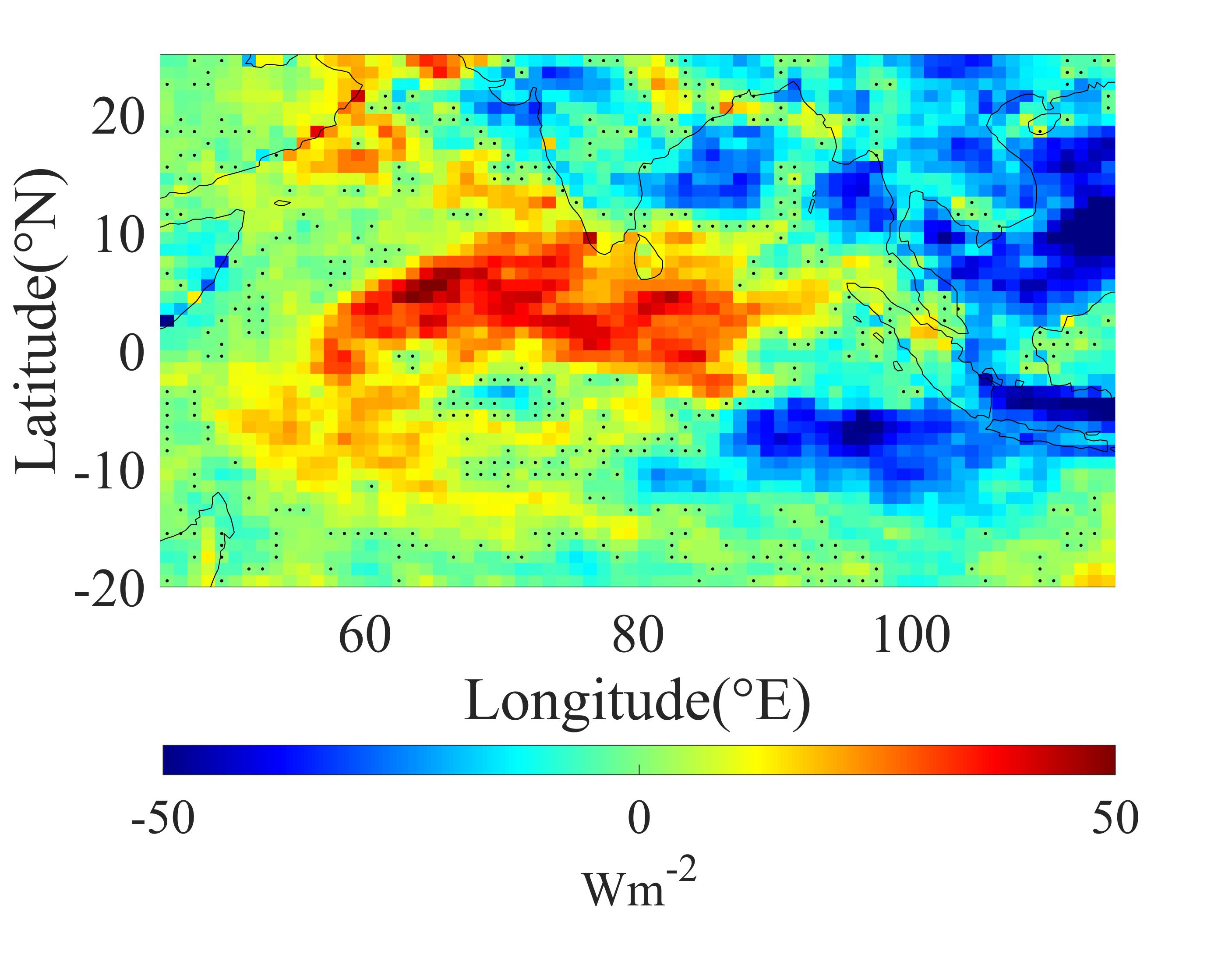

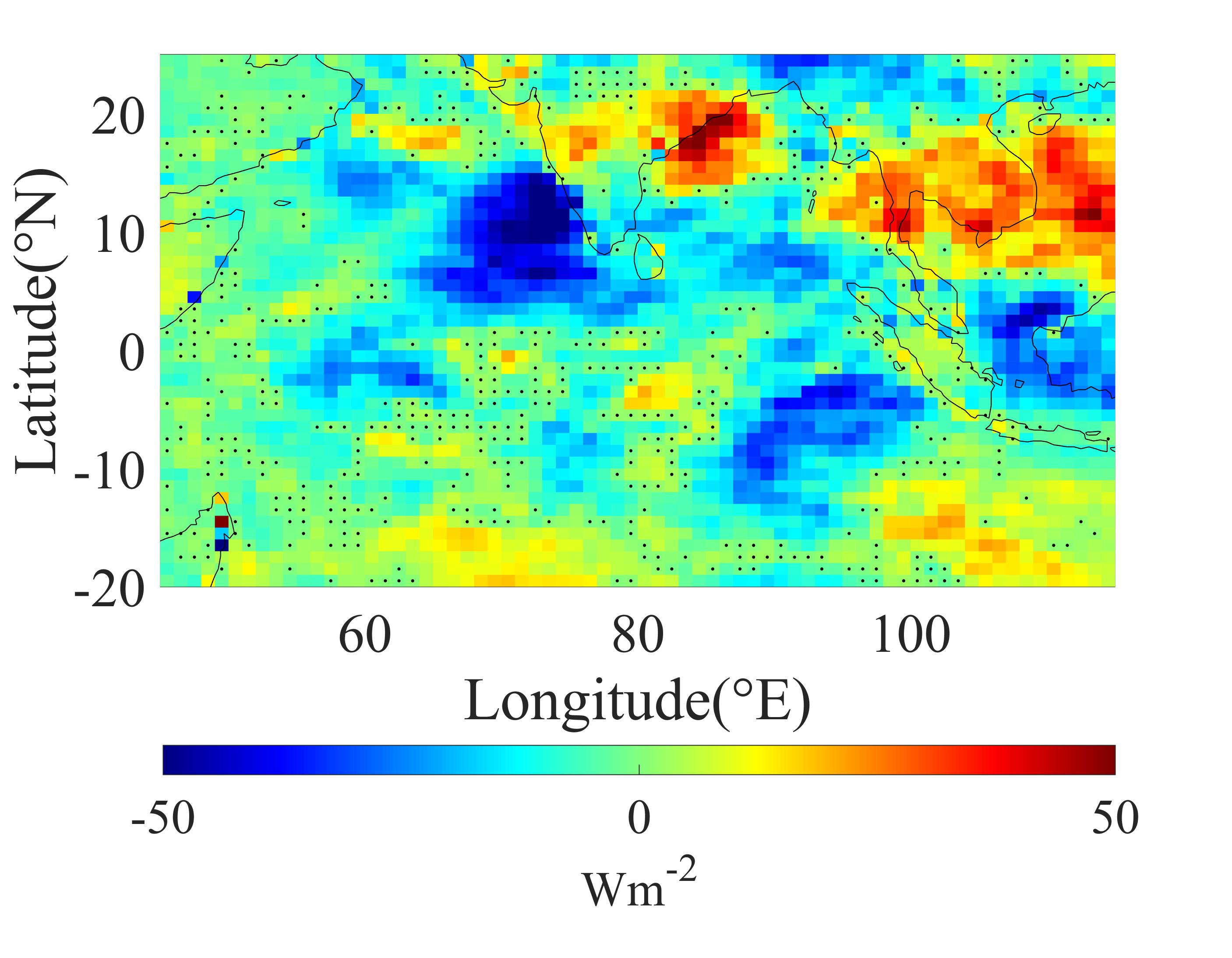

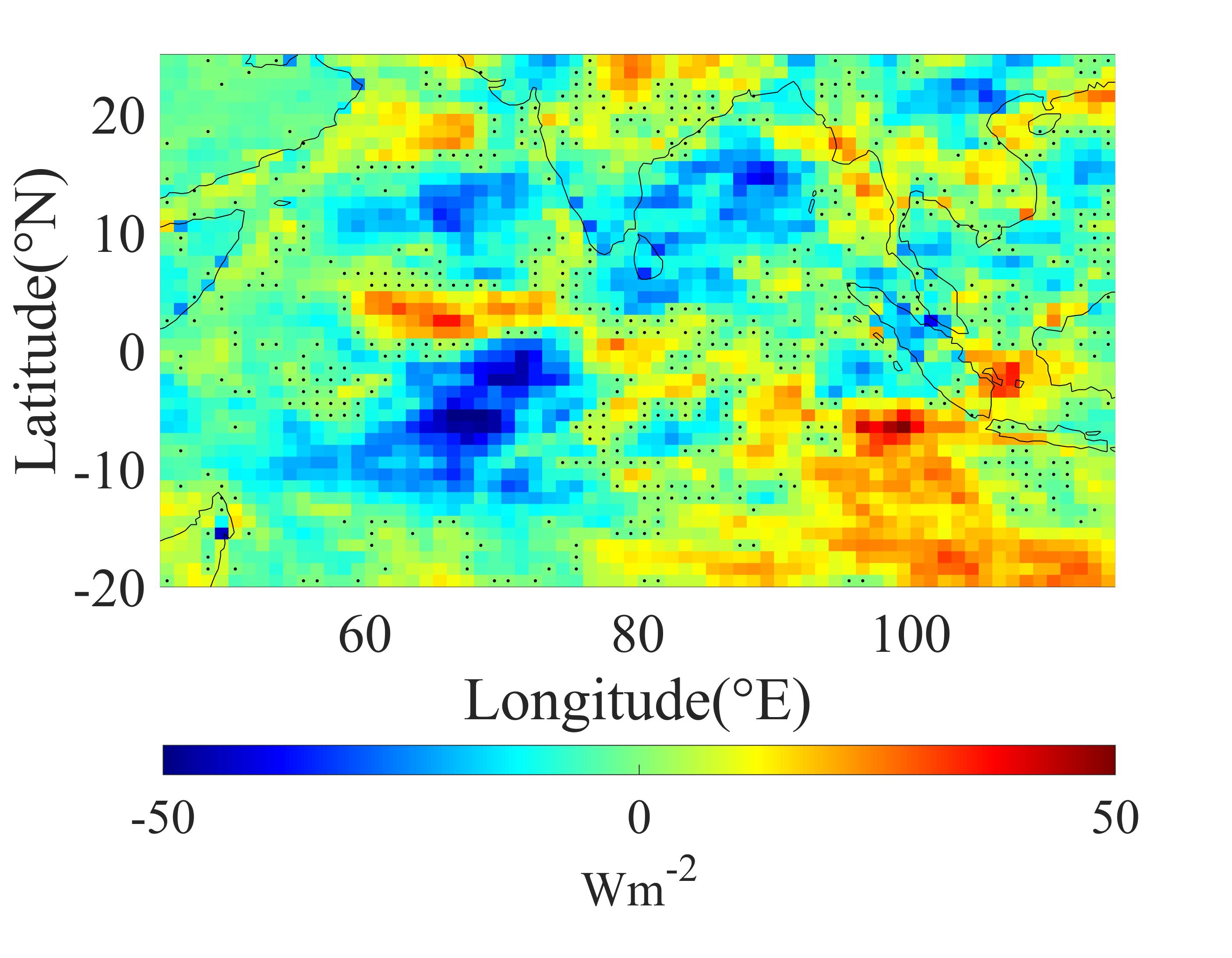

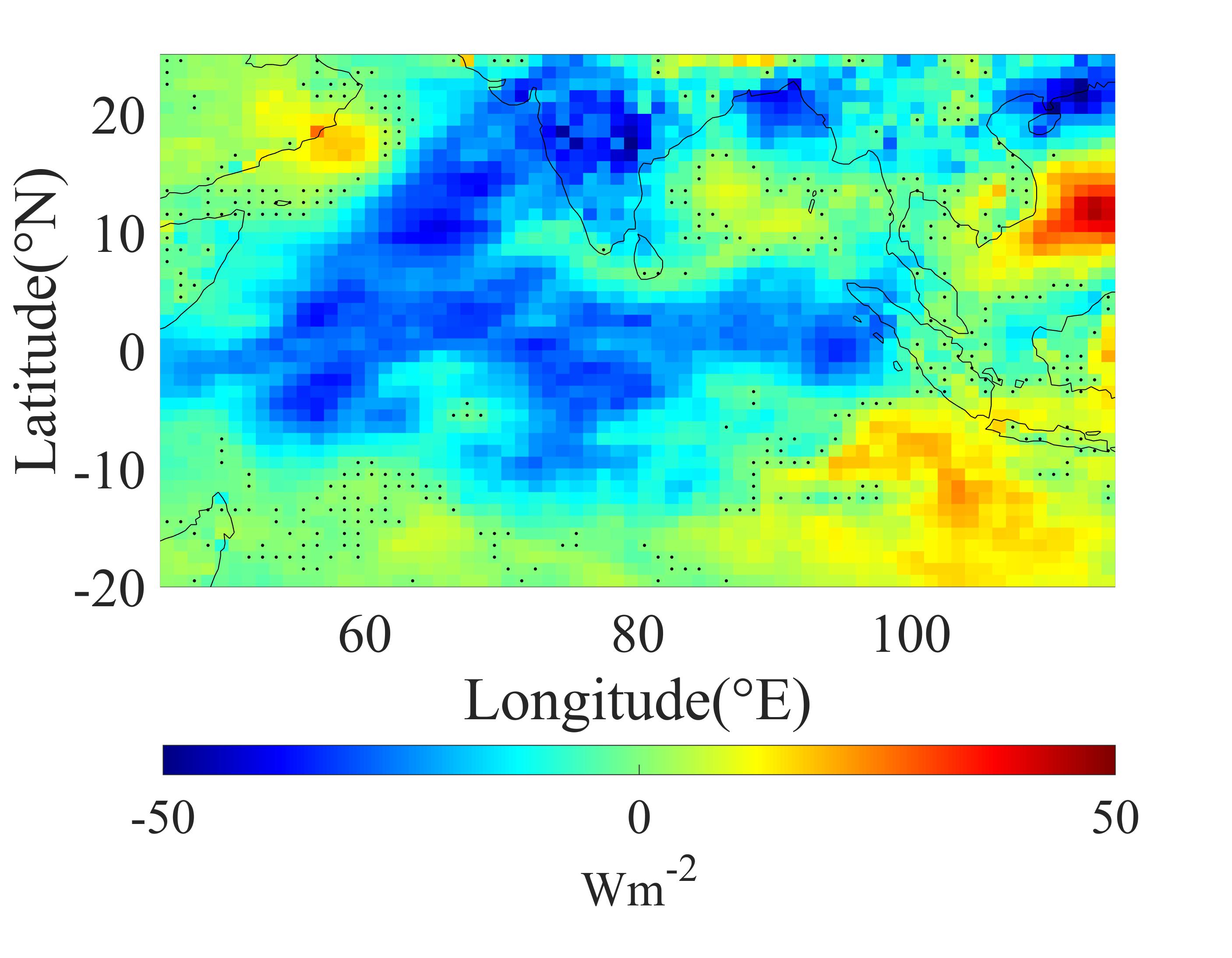

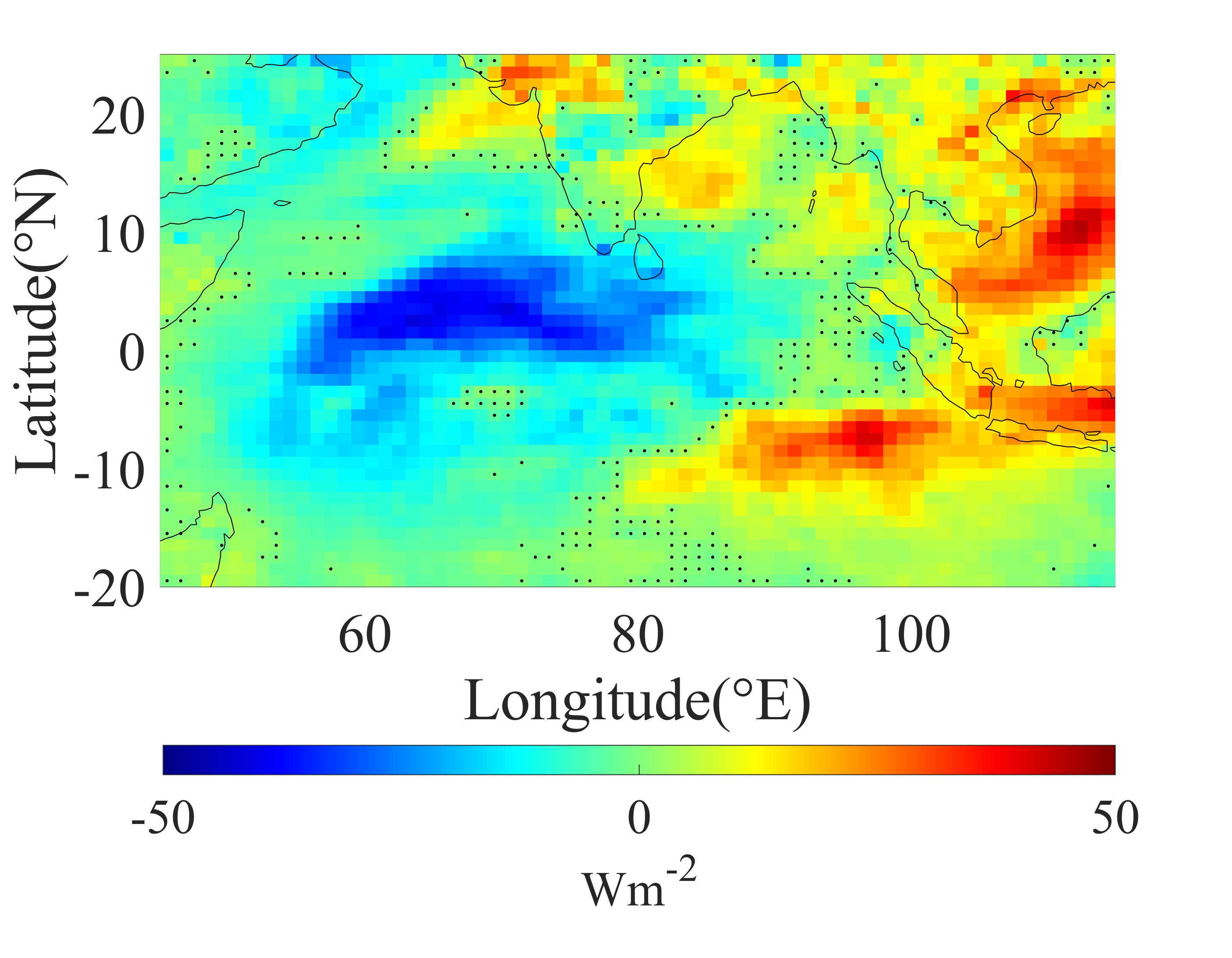

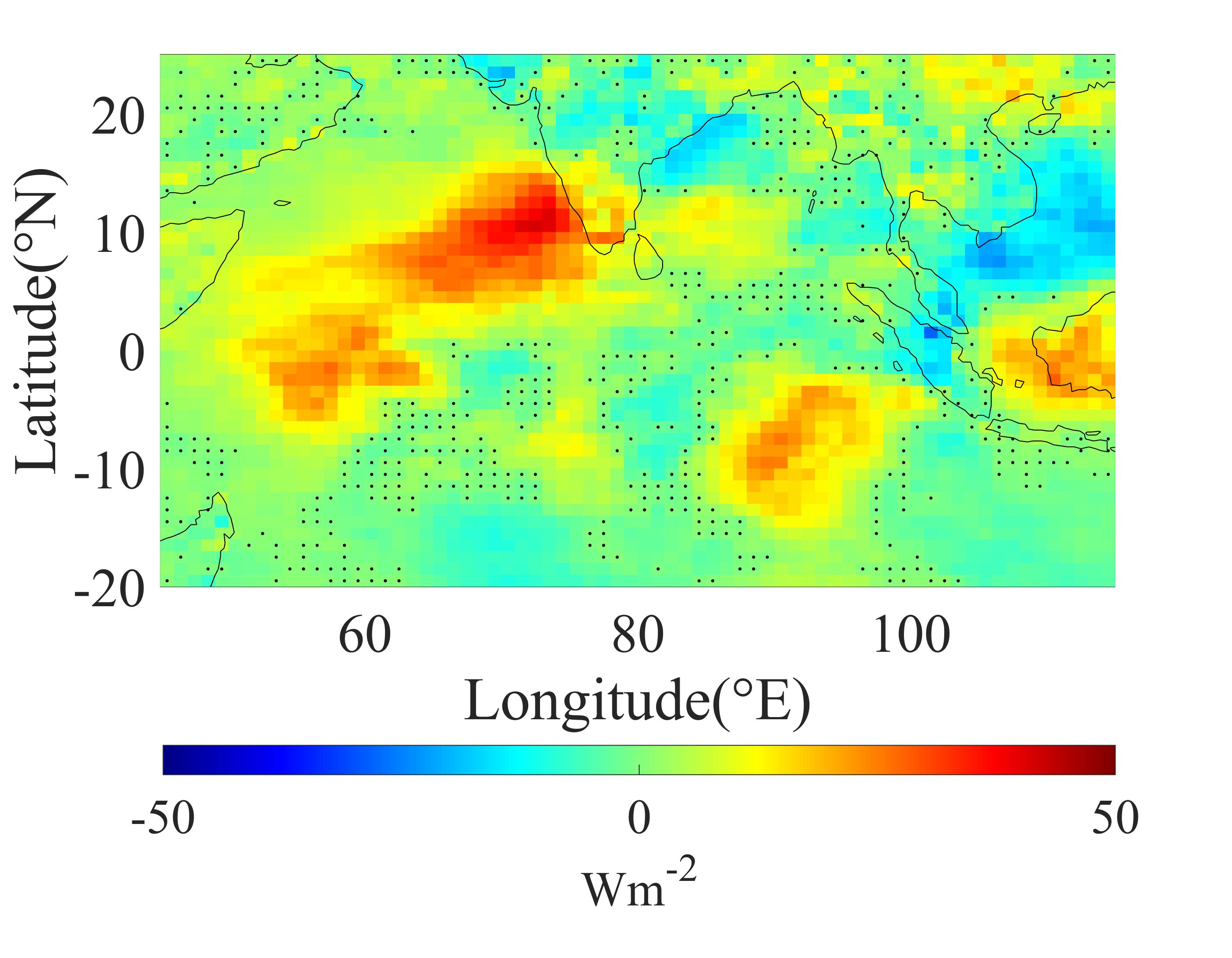

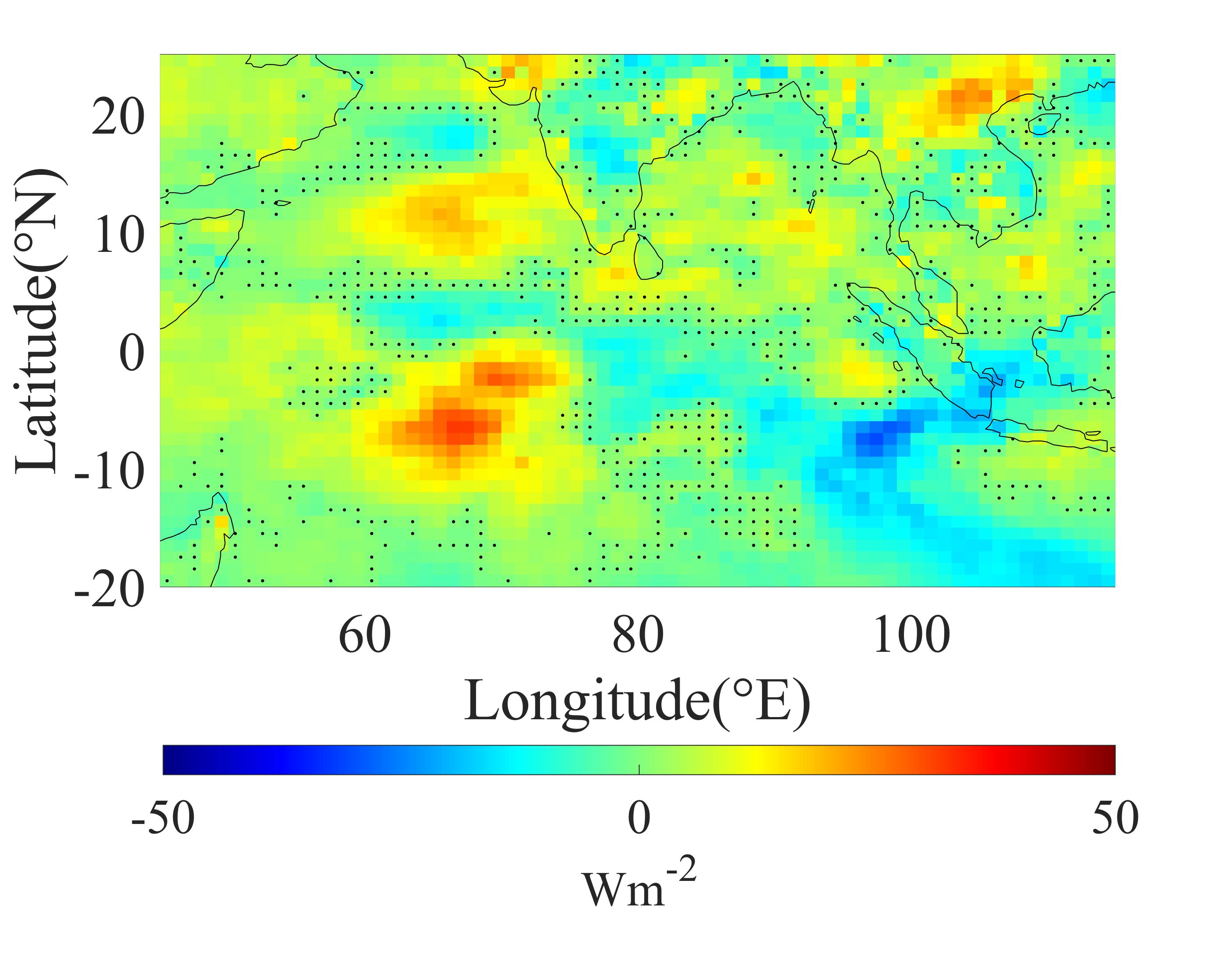

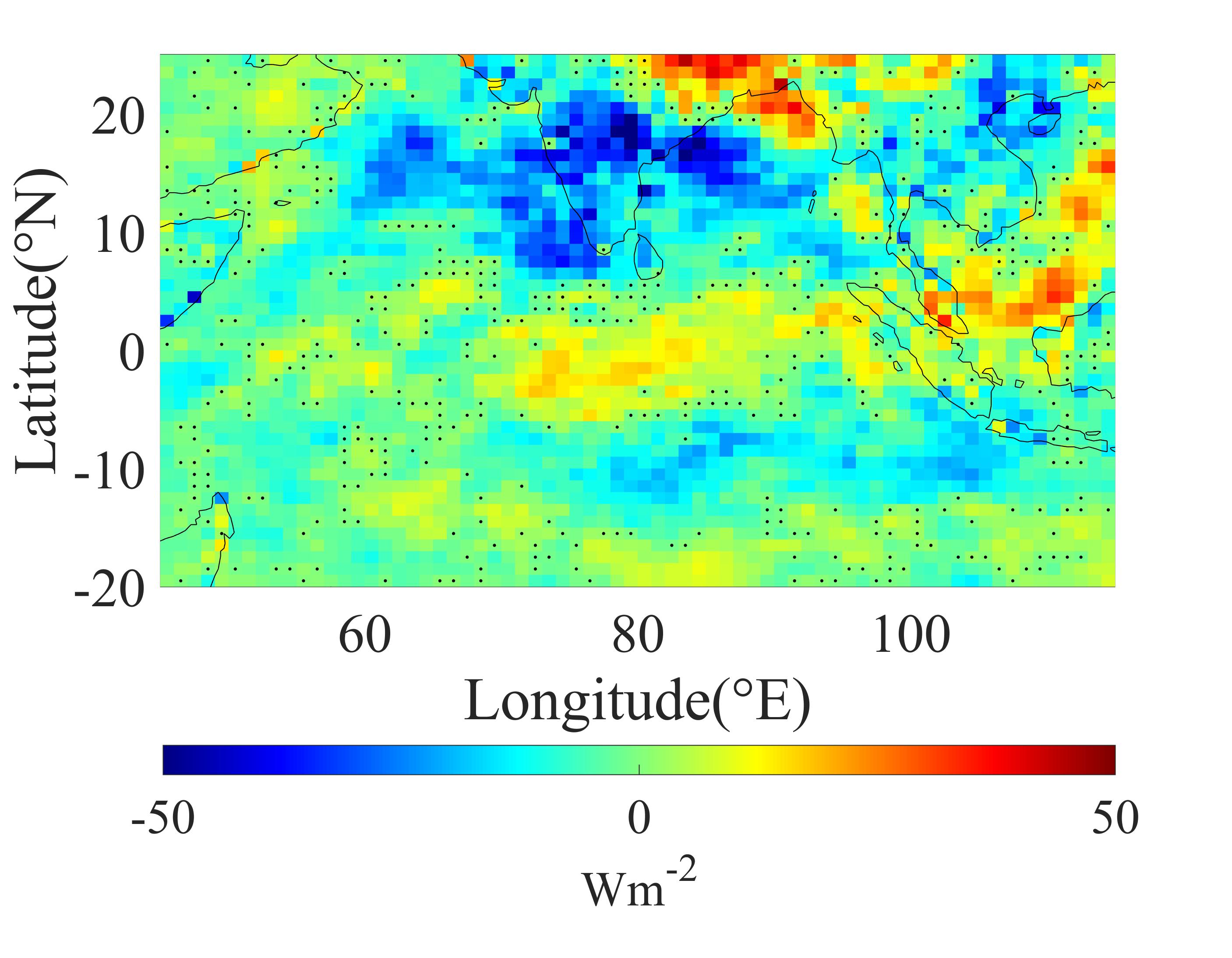

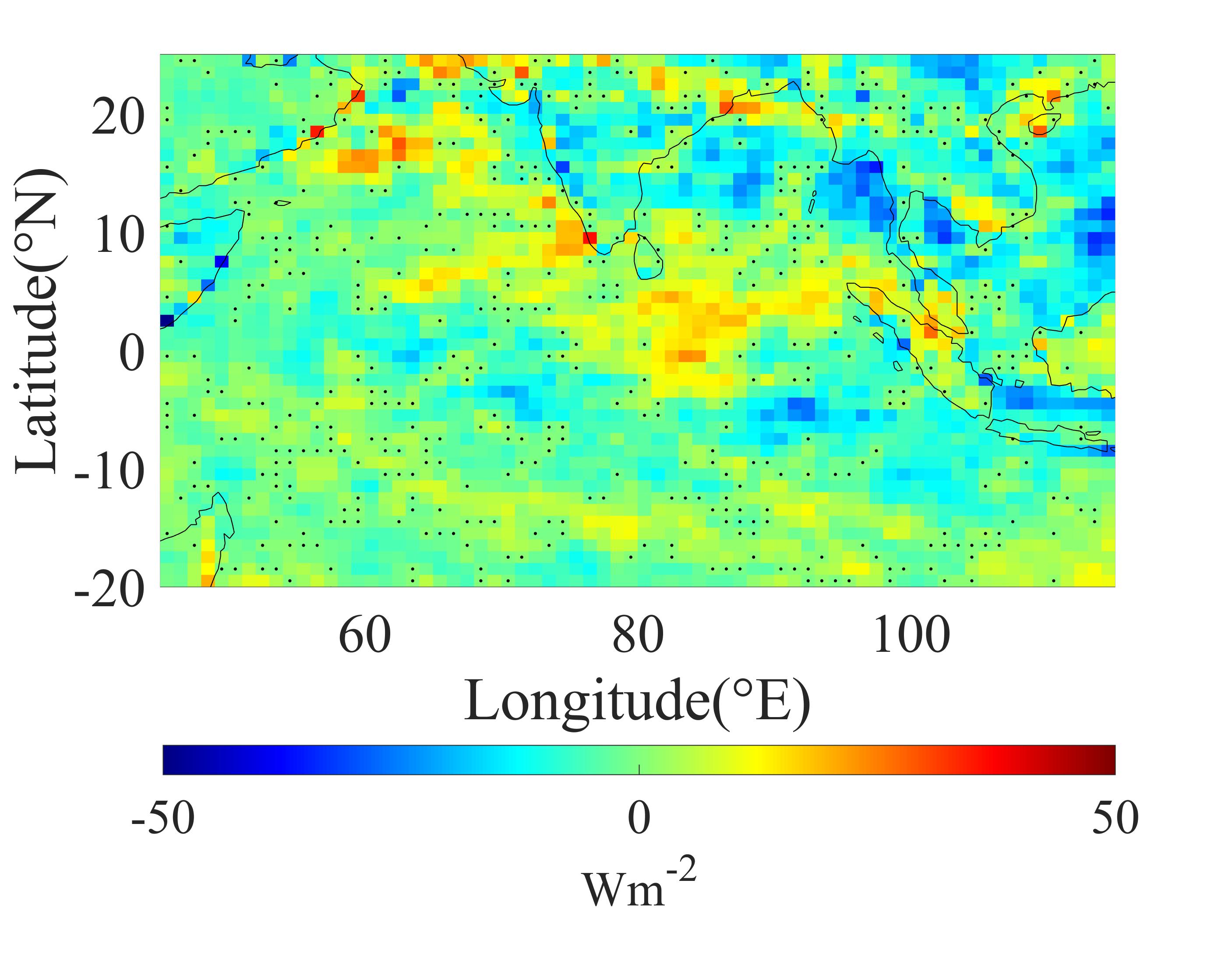

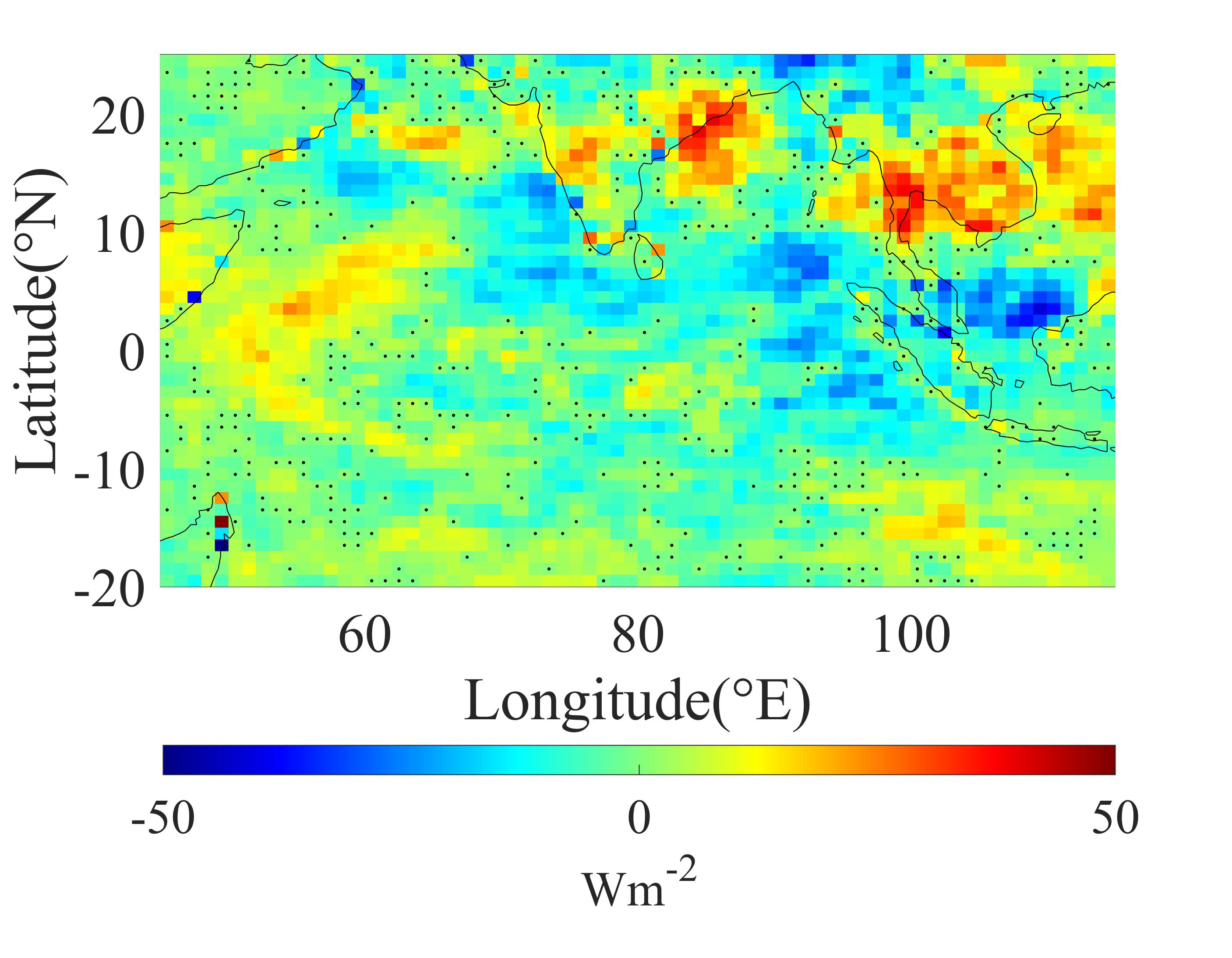

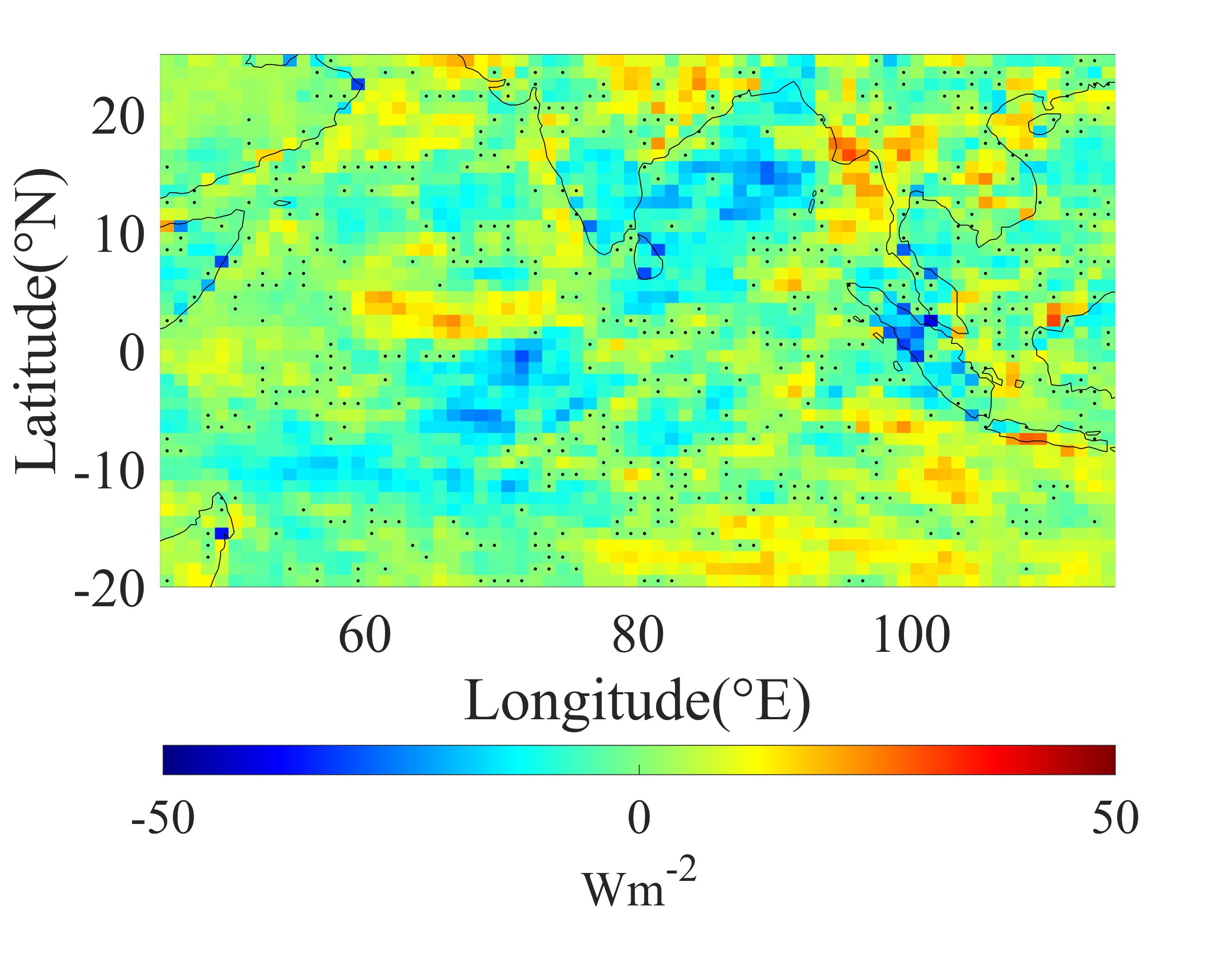


Changes in CRE_SW_ (left panel)_,_ CRE_LW_ (middle panel) and CRE_NET_ (right panel) in June (first row), July (second row), August (third row) and September (fourth row), during the period from 2000 to 2017. Total change during the period is computed by multiplying the annual trend with total number of years. Annual trends are estimated by excluding the years of El Niño and La Niña conditions. Black dots in the figures show the regions where the trends are not significant at 95% confidence level. The map is generated using MATLAB 2020a, www.mathworks.com.

**Fig. S12: Changes in CRE_SW,_ CRE_LW_ and CRE_NET_ during 2000 to 2017**

**Table S1: Mean and trends of CTH over NWIO and NEIO**

| Month | NWIO | | | NEIO | | |
| --- | --- | --- | --- | --- | --- | --- |
|  | Mean CTH (m)  (2000-2017) | Trend (myr^-1^) | %ΔCTH (2000-2017) | Mean CTH (m)  (2000-2017) | Trend (myr^-1^) | %ΔCTH (2000-2017) |
| Jun | 7357±24 | -69±3 | -16.88% | 9398±17 | -34±2 | -6.5% |
| Jul | 7198±25 | -44±3 | -11% | 9494±15 | 1±2 | 0.19% |
| Aug | 6458±26 | 106±2 | 29.54% | 9476±14 | 40±2 | 7.6% |
| Sep | 5594±25 | 37±1 | 11.91% | 9229±15 | 24±2 | 4.68% |
| %ΔCTH=(Trend × Number of Years)/ Mean CTH | | | | | | |

**Table S2: Statistics of CTH trend analysis**

|  | June | | | August | | |
| --- | --- | --- | --- | --- | --- | --- |
|  | Trend (ω)  myr^-1^ | 95% Confidence bounds (myr^-1^) | \|ω/σ_ω_\| | Trend (ω) | 95% Confidence bounds (myr^-1^) | \|ω/σ_ω_\| |
| Case 1 | -68.71±2.65 | (-179.6, 42.18) | 5.67 | 105.91±1.99 | (29.76, 182) | 17.16 |
| Case 2 | -105.87±2.53 | (-228.5, 16.74) | 9.02 | 107.62±1.94 | (27.41, 187.8) | 29.09 |
| Case 3 | -58.5±2.82 | (-197.4, 80.44) | 2.99 | 113.56±2.39 | (31.12, 196) | 14.82 |
| Case 4 | -113.26±2.85 | (-272.9, 46.39) | 5.32 | 122.89±2.82 | (37.62, 208.2) | 14.25 |
| Case 1: All Years Case 2: El Niño Years Excluded Case 3: La Niña Years Excluded  Case 4: El Niño and La Niña Years Excluded | | | | | | |

**Table S3: Changes in TOA fluxes over NWIO**

| Period | F_SW_  Clear Sky (Wm^-2^) | F_SW_  All Sky  (Wm^-2^) | F_LW_  Clear Sky (Wm^-2^) | F_LW_  All Sky (Wm^-2^) | ΔF_SW_ Clear Sky (Wm^-2^) | ΔF_SW_ All Sky  (Wm^-2^) | ΔF_LW_ Clear Sky (Wm^-2^) | ΔF_LW_ All Sky (Wm^-2^) |
| --- | --- | --- | --- | --- | --- | --- | --- | --- |
| JUN | 45.4 | 92.56 | 287.92 | 239.9 | 1.27±0.11 | -15.61±0.66 | 1.84±0.11 | 23.06±0.46 |
| JUL | 46.18 | 90.58 | 285.54 | 239.64 | -0.4±0.09 | -14.16±0.61 | 0.82±0.09 | 15.55±0.54 |
| AUG | 44.49 | 86.89 | 285.42 | 245.22 | 0.39±0.07 | 14.25±0.67 | -4.67±0.08 | -18.32±0.45 |
| SEP | 43.86 | 84.92 | 286.78 | 251.83 | -0.66±.05 | 5.27±0.63 | -1.34±0.11 | -6.82±0.4 |
| JUN-JUL | 45.79 | 91.57 | 286.73 | 239.77 | 0.44±0.07 | -14.89±0.05 | 1.33±0.07 | 19.31±0.43 |
| AUG-SEP | 44.18 | 85.91 | 286.1 | 248.52 | -0.13±0.04 | 9.76±0.45 | -3±0.07 | -12.57±0.29 |
| JUN-SEP | 44.98 | 88.74 | 286.41 | 244.14 | 0.15±0.04 | -2.56±0.37 | -0.84±0.06 | 3.37±0.27 |
| F_SW_ : Mean outgoing TOA shortwave flux during 2000-2017  F_LW_ : Mean outgoing TOA longwave flux during 2000-2017  ΔF_SW_ : Change in outgoing TOA shortwave flux during 2000-2017  ΔF_LW_ : Change in outgoing TOA longwave flux during 2000-2017 | | | | | | | | |

**Table S4: Changes in cloud radiative effects over NWIO**

| Period | CRE_SW_  (Wm^-2^) | CRE_LW_  (Wm^-2^) | CRE_NET_ (Wm^-2^) | ΔCRE_SW_  (Wm^-2^) | ΔCRE_LW_ (Wm^-2^) | ΔCRE_NET_ (Wm^-2^) |
| --- | --- | --- | --- | --- | --- | --- |
| JUN | -47.15±0.28 | 48.02±0.23 | 0.87±0.12 | 16.89±0.65 | -21.22±0.44 | -4.33±0.45 |
| JUL | -44.4±0.27 | 45.91±0.23 | 1.51±0.11 | 13.77±0.61 | -14.74±0.52 | -0.97±0.3 |
| AUG | -42.4±0.26 | 40.2±0.23 | -2.2±0.09 | -13.86±0.65 | 13.65±0.43 | -0.21±0.41 |
| SEP | -41.06±0.25 | 34.95±0.21 | -6.10±0.1 | -5.93±0.63 | 5.48±0.33 | -0.45±0.36 |
| JUN-JUL | -45.78±0.2 | 46.96±0.16 | 1.19±0.08 | 15.33±0.49 | -17.98±0.4 | -2.65±0.22 |
| AUG-SEP | -41.73±0.18 | 37.57±0.16 | -4.15±0.07 | -9.89±0.44 | 9.57±0.27 | -0.33±0.3 |
| JUN-SEP | -43.75±0.13 | 42.27±0.11 | -1.48±0.06 | 2.72±0.36 | -4.21±0.24 | -1.49±0.19 |
| CRE_SW,_ CRE_LW_ and CRE_NET_ are the mean shortwave, longwave and net cloud radiative effects respectively, during 2000-2017.  ΔCRE_SW_, ΔCRE_LW_ and ΔCRE_NET_ are the changes in shortwave, longwave and net cloud radiative effect respectively, during 2000-2017. | | | | | | |
